# Lifetime over 10000 hours for organic solar cells with Ir/IrO_x_ electron-transporting layer

**DOI:** 10.1038/s41467-023-36937-8

**Published:** 2023-03-04

**Authors:** Yanxun Li, Bo Huang, Xuning Zhang, Jianwei Ding, Yingyu Zhang, Linge Xiao, Boxin Wang, Qian Cheng, Gaosheng Huang, Hong Zhang, Yingguo Yang, Xiaoying Qi, Qiang Zheng, Yuan Zhang, Xiaohui Qiu, Minghui Liang, Huiqiong Zhou

**Affiliations:** 1 CAS Key Laboratory of Nanosystem and Hierarchical Fabrication, National Center for Nanoscience and Technology, Beijing, 100190 P. R. China; 2 Center of Materials Science and Optoelectronics Engineering, University of Chinese Academy of Sciences, Beijing, 100049 China; 3 School of Chemistry, Beijing Advanced Innovation Center for Biomedical Engineering, Beihang University, Beijing, 100191 P. R. China; 4 CAS Key Laboratory of Standardization and Measurement for Nanotechnology, National Center for Nanoscience and Technology, Beijing, 100190 P. R. China; 5 Shanghai Synchrotron Radiation Facility (SSRF), Zhangjiang Lab, Shanghai Advanced Research Institute, Chinese Academy of Sciences, Shanghai, 201204 China; 6 Present Address: Center of Materials Science and Optoelectronics Engineering, University of Chinese Academy of Sciences, Beijing, 100049 China

**Keywords:** Materials for devices, Nanoscale materials, Solar cells, Nanoparticles

## Abstract

The stability of organic solar cells is a key issue to promote practical applications. Herein, we demonstrate that the device performance of organic solar cells is enhanced by an Ir/IrO_x_ electron-transporting layer, benefiting from its suitable work function and heterogeneous distribution of surface energy in nanoscale. Notably, the champion Ir/IrO_x_-based devices exhibit superior stabilities under shelf storing (*T*_80_ = 56696 h), thermal aging (*T*_70_ = 13920 h), and maximum power point tracking (*T*_80_ = 1058 h), compared to the ZnO-based devices. It can be attributed to the stable morphology of photoactive layer resulting from the optimized molecular distribution of the donor and acceptor and the absence of photocatalysis in the Ir/IrO_x_-based devices, which helps to maintain the improved charge extraction and inhibited charge recombination in the aged devices. This work provides a reliable and efficient electron-transporting material toward stable organic solar cells.

## Abstract (web summary)

Long-term stability of organic solar cells is critical to promote practical applications. Here, the authors utilize iridium/iridium oxide nanoparticles as the electron-transporting material and realize enhanced device stabilities under thermal aging with T70 of over 10000 h.

## Introduction

During the past several years, the power conversion efficiency (PCE) of organic solar cells (OSCs) has been enhanced rapidly^1–3^ with the development of polymer donors and non-fullerene acceptors^4^. Currently, improving the lifetime of OSCs is the top priority to promote practical applications^5–9^. Except for the material and morphology of the active layer^10–12^, the hole/electron-transporting layer (HTL/ETL)^13,14^ and device structure^15^ also determine the stability. The inverted structure has been proven to be a feasible strategy for achieving superior device stability^15^. Traditional metal oxides (such as TiO_x_^16,17^ and ZnO^5,14^) used as the electron-transporting materials in inverted OSCs usually suffer from unfavorable light-soaking^18^ or photocatalysis problem^14^. Efforts have been devoted to seeking reliable cathode interfacial materials, including nonconjugated electrolytes (such as PEI^19^ and PEIE^20^), conjugated polyelectrolytes (such as PFN-Br^21^ and PDNIT-F3N^22^), and conjugated small-molecular electrolytes (such as PDINO^23^ and PDINN^24,25^), etc. However, the long-term stability of OSCs is still unsatisfactory, and stable electron-transporting materials are urgently needed.

In this contribution, we provide a reliable and efficient electron-transporting material for stable OSCs. Iridium/Iridium oxide (Ir/IrO_x_) nanoparticles are synthesized through the solution process and utilized as an electron-transporting material in OSCs. The enhanced PCE of Ir/IrO_x_-based devices compared with one of the ZnO-based devices originates from its suitable work function, the regulation of the optical field, and the heterogeneous surface energy distribution in the nanoscale (HeD-SE). Benefiting from the well-organized and stable bulk-heterojunction (BHJ) film and the absence of photocatalysis, the Ir/IrO_x_-based devices exhibit excellent long-term stability under shelf storing (*T*_80_ = 56,696 h vs. 12,075 h, which is the time when the PCE of the device decreases to the 80% of initial PCE), thermal aging (*T*_70_ = 13,920 h vs. 2198 h, the time when the PCE of the device decreases to the 70% of initial PCE), and maximum power point (MPP) tracking (*T*_80_ = 1058 h vs. 586 h) compared with the ZnO-based devices. The impact of Ir/IrO_x_ on the morphological evolution of BHJ films is further discussed in detail to explore the degradation mechanism. Moreover, the stability of OSCs under thermal circulation and ultraviolet (UV)-irradiation are also improved by the Ir/IrO_x_ ETL, which illustrates that the possibility of operating under extreme environments can be increased by applying Ir/IrO_x_.

## Results

### Characterization of Ir/IrO_x_

**Fig. 1:**
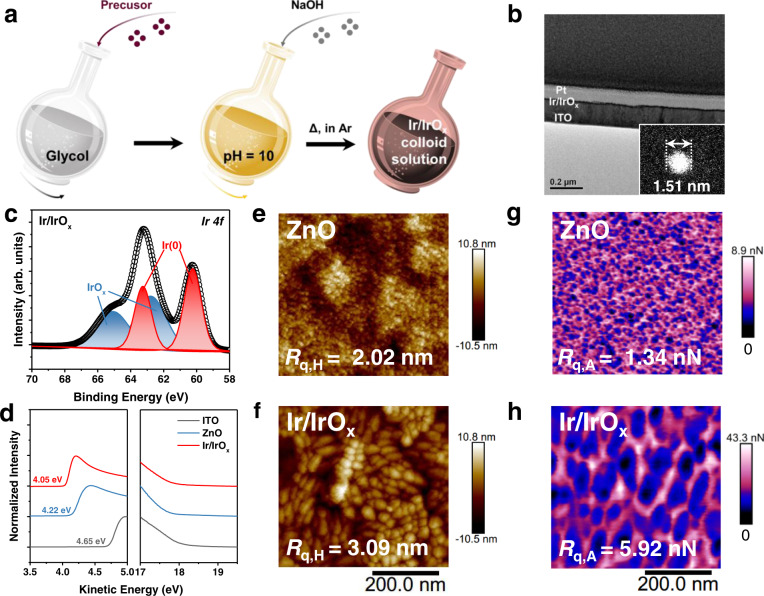
Characterization of Ir/IrO_x_. **a** The synthesis process of Ir/IrO_x_ nanoparticles. In the synthesis route, the Δ represents heating at 160 °C. **b** The cross-sectional transmission electron microscope (TEM) patterns of Ir/IrO_x_ samples spin-coated on ITO, which was prepared by a focused ion beam (FIB). The insert is the high-resolution image of a single Ir/IrO_x_ nanoparticle on copper mesh, which was characterized by a special aberration-corrected transmission electron microscope (AC-TEM). **c** The *Ir 4f* X-ray photoelectron spectroscopy (XPS) plot of Ir/IrO_x_ nanoparticles. **d** The ultraviolet photoelectron spectroscopy (UPS) characterizations of ITO (gray), ZnO (blue), and Ir/IrO_x_ (red). The Peak-force quantitative nanomechanical mappings **(**PFQNM) morphology (**e, f**) and adhesion (**g, h**) patterns of ZnO and Ir/IrO_x_ films.

In this study, Ir/IrO_x_ nanoparticles have been selected as an electron-transporting material since iridium (Ir) -based nanoparticles exhibit excellent charge-transfer ability in electrocatalysis^26–29^. The Ir/IrO_x_ nanoparticles were prepared through the mild colloid solution method^30,31^ with IrCl_3_·3H_2_O precursor under Ar atmosphere (Fig. 1a and Supplementary Fig. 1). Compared with the ZnO precursor solution, the colloidal solution of iridium-based nanoparticles shows better stability after storing in the air for 2 years (Supplementary Fig. 2). The mean diameter of the nanoparticle is 1.50 ± 0.30 nm measured by the special aberration-corrected transmission electron microscope (AC-TEM) (Fig. 1b and Supplementary Fig. 3). The cross-sectional TEM patterns (Fig. 1b and Supplementary Fig. 4) demonstrate that Ir/IrO_x_ nanoparticles can be deposited on the ITO substrate to form a condensed and uniform film, whose mean thickness is 4.66 ± 0.37 nm (Supplementary Fig. 5).

The X-ray photoelectron spectroscopy (XPS) characterization assisted to determine the oxidation state of nanoparticles (Supplementary Fig. 6). In the *Ir 4* *f* core-level spectrum (Fig. 1c), two groups of peaks located at 60.23/63.26 eV and 62.79/65.24 eV are attributed to the metallic Ir and IrO_x_^32,33^, respectively. Energy level matching is an important basis for selecting an ETL. By characterizations of ultraviolet photoelectron spectroscopy (UPS, Fig. 1d), the work function (WF) of the Ir/IrO_x_ and ZnO were determined to be 4.05 eV and 4.22 eV, respectively. Besides, the WFs of the bare ITO and ITO modified by glycol (solvent), NaOH in glycol (pH = 10), and IrCl_3_ (precursor) were found to be 4.65 eV, 4.44 eV, 4.27 eV, and 4.27 eV, respectively (see Fig. 1d and Supplementary Fig. 7). The gradient changes exclude the effect of pure solvent and other possible impurities. Meantime, the result of UPS characterization also confirms the successful deposition of Ir/IrO_x_ nanoparticles on top of ITO substrates. The shallower WF of Ir/IrO_x_ indicates that the Ir/IrO_x_ nanoparticles can further minimize the interfacial barrier between the BHJ film and electrode^34^, which improves the charge extraction and carrier transport in inverted devices compared with the ZnO ETL.

To obtain more information about the surface of interlayers, the Peak-force quantitative nanomechanical mappings (PFQNM) characterization was performed. In Fig. 1e, f and Supplementary Fig. 8, the root-mean-square roughness of morphology (*R*_q,H_) of the bare ITO, ZnO, and Ir/IrO_x_ in topographic patterns are 3.50 nm, 2.02 nm, and 3.09 nm, respectively, which indicates that the modification of ZnO and Ir/IrO_x_ can decrease the roughness. A more heterogeneous surface energy distribution in the nanoscale (HeD-SE)^35^ is observed at the surface of Ir/IrO_x_ (Fig. 1h). The root-mean-square roughness of adhesion (*R*_q,A_) parameters of bare ITO, ZnO, and Ir/IrO_x_ are 2.84 nN, 1.34 nN, and 5.92 nN, respectively (Fig. 1g, h, Supplementary Fig. 8 and Supplementary Table 1). Simultaneously, the surface energy (*γ*_s_) of the ZnO and the Ir/IrO_x_ calculated from contact angle measurement are 66.39 mN/m and 76.23 mN/m (Supplementary Fig. 9). The heterogeneous surface energy distribution and the improved *γ*_s_ in bottom interlayers could further regulate the morphology of upper BHJ layers and assist the formation of BHJ film with well-organized stacking and phase separation, which should be beneficial to the resulted device performance^35,36^.

### Device performance

**Fig. 2:**
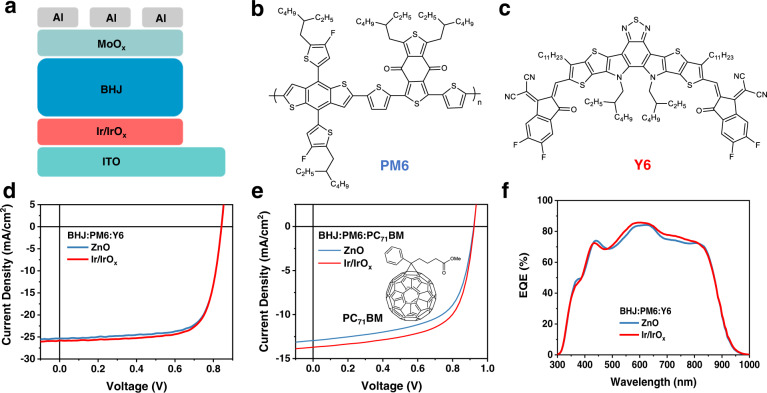
Device performance. **a** The device structure is based on Ir/IrO_x_ nanoparticles. Molecular structures of PM6 (**b**) and Y6 (**c**). *J-V* characteristics of PM6:Y6 (**d**) and PM6:PC_71_BM (**e**) devices on different electron-transporting materials. The insert in **e** is the molecular structure of PC_71_BM. **f** The external quantum efficiency (EQE) curves of PM6:Y6 devices based on ZnO and Ir/IrO_x_.

Inverted OSCs were fabricated based on the device structure of ITO/Ir/IrO_x_ nanoparticles/BHJ/MoO_x_/Al (Fig. 2a). After optimization (Supplementary Fig. 10 and Supplementary Tables 2–4), the best PCE of the Ir/IrO_x_-based device is improved to 15.89% (Fig. 2d and Supplementary Table 5), when compared to the one (15.58%) of the ZnO-based device in PM6:Y6 cells (Fig. 2b, c), and 8.11% in PM6:PC_71_BM (vs. 7.38% with ZnO) and 16.19% in PM6:Y6:PC_71_BM (vs. 15.95% with ZnO) (Fig. 2e, Supplementary Fig. 11 and Supplementary Table 5). The results illustrate the superior interfacial characteristic and universality of Ir/IrO_x_ ETLs to improve the device performance in OSCs. As seen from the results shown in Supplementary Fig. 12 and Supplementary Table 6, the poor performance of the devices prepared using bare ITO and ITO modified by pure solvent or possible impurities (glycol, NaOH, and IrCl_3_) rules out the solvent or impurity effects and highlights the positive role of Ir/IrO_x_ in improving the efficiency. We further chose PM6:Y6 device as a model system to study the origin of the enhanced PCEs of Ir/IrO_x_-based devices compared with the ZnO-based devices. The enhancement of PCEs mainly comes from enlarged *J*_sc_ in the Ir/IrO_x_-based device which can be confirmed by external quantum efficiency (EQE) measurements with an improved response from 450 nm to 850 nm (Fig. 2f).

### Device physics

**Fig. 3:**
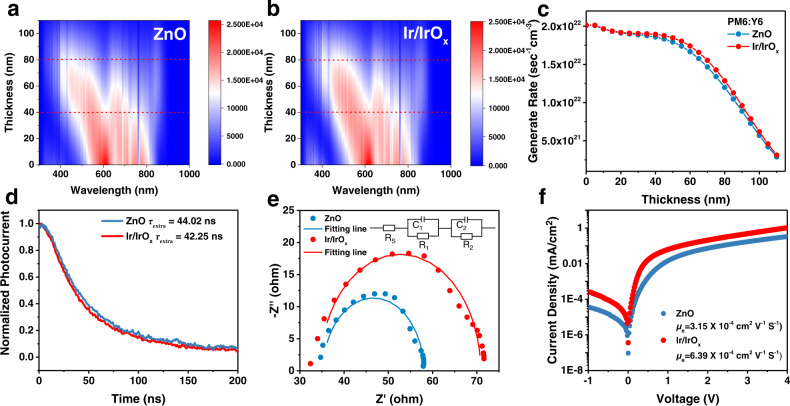
Device physics. The optical simulation for the exciton generation rate profiles of BHJ films in the (**a**) ZnO- and (**b**) Ir/IrO_x_-based OSCs. **c** The plot of exciton generation rate with the depth of active layer extracted from optical simulations. Transient photocurrent (TPC) plots (**d**) and electrochemical impedance spectroscopy (EIS) (**e**) of PM6:Y6 devices based on ZnO and Ir/IrO_x_ ETLs. In the equivalent circuit of EIS, the R_s_ represents the series resistance and R_1_ and R_2_ are shunt resistances. The capacitors are represented by C_1_ and C_2_. **f** Dark current curves of single-electron PM6:Y6 devices based on ZnO and Ir/IrO_x_ ETLs, respectively.

Concerning the difference in the optical transmittance and thickness of Ir/IrO_x_ and ZnO (Supplementary Figs. 4, 5, 13, 14), the effect of the ETLs on the distribution of optical field in devices was simulated through the transfer-matrix formalism method^37^. In Fig. 3a–c and Supplementary Fig. 15, the optical electric field intensity and exciton generation rate in PM6:Y6 film on Ir/IrO_x_ nanoparticles are higher than the film on ZnO, which can result in the higher *J*_sc_. It also demonstrates that the Ir/IrO_x_ ETL can act as an optical spacer and optimize the distribution of optical field in the devices^38^.

Further, we explored the impact of Ir/IrO_x_ on charge extraction, recombination, and transport. By transient photocurrent characterization (TPC, Fig. 3d), the charge extraction time (*τ*_extra_) of devices based on ZnO and Ir/IrO_x_ were determined to be 44.02 ns and 42.25 ns, which demonstrates that the charge extraction is accelerated by using Ir/IrO_x_ to replace the ZnO film. In the *P*_light_-*J*_sc_ characterization (Supplementary Fig. 16a), the *J*_sc_ exhibits a power-law dependence as ~*P*_light_^*α*^ and the value of *α* for the devices with ZnO and Ir/IrO_x_ are 0.96 and 0.97, respectively, which demonstrates the dominance of bimolecular charge recombination at short-circuit conditions^39,40^. Besides, the slopes of *V*_oc_ versus *P*_light_ curves were identified to be 1.31*k*_B_T/q and 1.27*k*_B_T/q for the PM6:Y6 devices with ZnO and Ir/IrO_x_ ETLs, respectively (Supplementary Fig. 16b). Because the upper deviation of the slope from *k*_B_T/q means the role of trap-assisted (Shockley Read-Hall, SRH) charge recombination^40^, the result of *P*_light_-*V*_oc_ confirms that the SRH recombination can be inhibited by Ir/IrO_x_.

In the Nyquist plots of electrochemical impedance spectroscopy (EIS, Fig. 3e), the Ir/IrO_x_-based devices exhibit a larger recombination resistance (*R*_rec_, 36.69 Ω) than the *R*_rec_ of the ZnO-based devices (22.32 Ω), confirming that the recombination is suppressed by Ir/IrO_x_^41^. Meantime, in the Bode plots of EIS (Supplementary Fig. 17) the characteristic frequency peaks (*f*_max_) are located at 2.13$\times$10^5 ^Hz for ZnO-based devices and 2.61$\times$10^5 ^Hz for Ir/IrO_x_-based devices, respectively. Given that the charge transport time constant (*τ*) of interface capacitance can be defined by the relation: *f*_max_ ∝ 1/*τ*^42^, the devices based on Ir/IrO_x_ exhibit a smaller value of *τ*, which illustrates that the Ir/IrO_x_ can assist to achieve faster charge transport.

Then, to explore the effect of different electron-transporting materials on the transport properties in the photoactive layer, electron-only devices (structure: ITO/ZnO or Ir/IrO_x_/BHJ/PNDIT-F3N/Al) were fabricated. The electron mobility (*μ*_e_) of BHJ layer was assessed by fitting the dark current of the electron-only devices (see Fig. 3f) with the well-established Mott-Gurney law^43^. The *μ*_e_ is determined to be 6.39 × 10^−4^ cm^2^ V^−1^ s^−1^ in the Ir/IrO_x_ devices, while a lower *μ*_e_ (3.15 × 10^−4^ cm^2^ V^−1^ s^−1^) is found in the ZnO-based device. As indicated by the enlargement of mobility, the promoted electron transport could be the merits of the optimized morphology of the BHJ film in conjunction with the improved charge injection in the presence of Ir/IrO_x_.

In short, the above results confirm that the Ir/IrO_x_ layer is an excellent optical spacer and a competitive ETL to optimize the distribution of optical field, charge extraction, recombination, and transport. At the same time, the superior charge behavior is also related to the improved molecular stacking of BHJ films which is optimized by the heterogeneous nanoscale surface energy distribution of Ir/IrO_x_ nanoparticles.

### Device stability

**Fig. 4:**
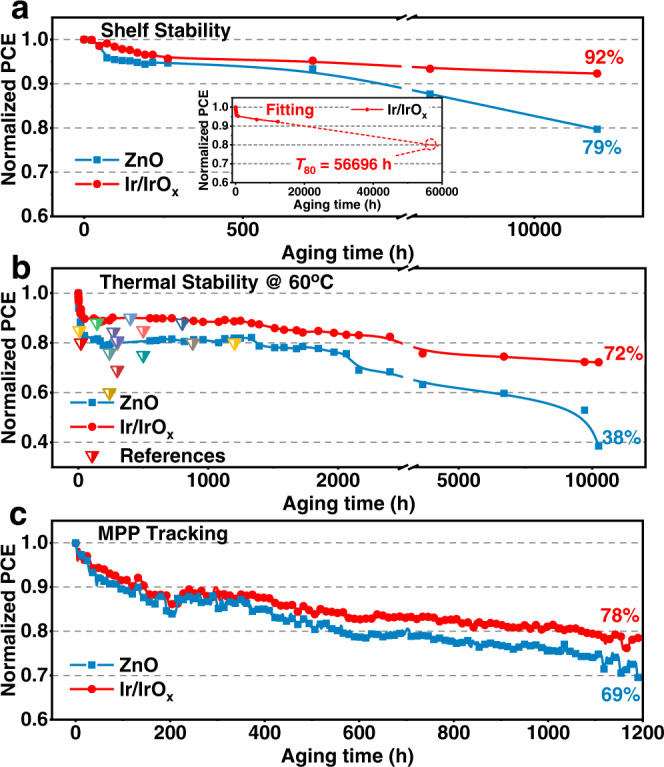
Device stability. PCE evolution plots of champion devices in the stability test of shelf storing (**a**), thermal aging (**b**), and maximum power point (MPP) tracking under 100 mW/cm^2^ illumination (**c**). The insert graph in Fig. 3a is the corresponding fitting line of PCE evolution plot of champion Ir/IrO_x_-based device in shelf stability. Triangles in Fig. 3b are the thermal-stability data summarized from references.

The long-term stability of OSCs is one of the key factors restricting their practical application^44,45^. To explore the effect of Ir/IrO_x_ on the stability of devices, the stability of devices under shelf storing, thermal aging, and operating at MPP were characterized sequentially. Two essential parameters of *T*_80_ and *T*_70_ (the time when the PCE of the device respectively decreases to 80% and 70% of the initial PCE) were applied to examine the stability of devices on different ETLs. In Fig. 4a, Table 1, Supplementary Fig. 18, 19 and Supplementary Table 7, the PM6:Y6 devices with Ir/IrO_x_ possess more excellent shelf stability, whose champion device still keeps 92% of the initial PCE after 12,000 h storing. The fitting *T*_80_ of the champion device with Ir/IrO_x_ ETL reaches 56,696 h (approaching 6.5 years, in Table 1) and the averaged *T*_80_ value reaches 52,489 h (Supplementary Table 8). While, the shelf stability *T*_80_ of the best ZnO-based device is only 12,075 h (averaged *T*_80_ is 11391 h), which is far behind the Ir/IrO_x_-based device.

**Table 1:** The statistic of lifetime

ETLs	Aging condition	*T*_80_ (h)	*T*_70_ (h)
ZnO	Shelf stability ^a^	12075	-
	Thermal aging ^b^	1366	2198
	MPP tracking ^c^	586	1188
Ir/IrO_x_	Shelf stability ^a^	56696 ^d^	-
	Thermal aging ^b^	2800	13920 ^d^
	MPP tracking ^c^	1058	2007 ^d^

Summary of *T*_80_ and *T*_70_ data extracted from the stability test of champion devices under different aging conditions.

^a^Based on devices stored in an N_2_-filled glovebox to track shelf stability.

^b^Based on devices placed on a high-precision hotplate at 60 °C.

^c^Based on devices placed under 100 mW/cm^2^ illumination with LED light operated at MPP.

^d^The time was obtained by fitting the PCE evolution plot of the champion device with Ir/IrO_x_ ETL.

Then, the impact of electron-transporting materials on the device stability during thermal aging was studied. In Fig. 4b, Table 1 and Supplementary Fig. 20, the PCE of the champion PM6:Y6 device with Ir/IrO_x_ ETL maintains 80% of the initial value after 2800 h. By contrast, the PCE of the ZnO-based device reduces to 67% of the initial efficiency. After heating for 10000 h, the PCE of the champion device with Ir/IrO_x_ ETL still keeps 72% of the initial value and the *T*_70_ reaches 13920 h (averaged *T*_70_ is 10248 h, see Table 1, Supplementary Table 8 and Supplementary Fig. 20), which is the longest *T*_70_ lifetime reported so far to the best of our knowledge^5,46–55^. Regarding the control device with ZnO ETL, the *T*_70_ is only 2198 h, which is much lower than the Ir/IrO_x_-based device.

We further tracked the device stability operated at MPP under 100 mW/cm^2^ illumination with LED light^56^. As shown in Supplementary Fig. 21, compared with the mean *T*_80_ of the PM6:Y6 device with ZnO ETL (248 h), the Ir/IrO_x_-based device exhibits a longer mean *T*_80_ (513 h). After the involvement of the third component of PC_71_BM into the active layer^4,51^, the stability of PM6:Y6:PC_71_BM-based OSCs with Ir/IrO_x_ nanoparticles has been improved, exhibiting a champion *T*_80_ of 1058 h and a *T*_70_ of 2007 h (Fig. 4c, Table 1 and Supplementary Fig. 22), which are much longer than the best *T*_80_ and *T*_70_ of the ZnO-based device (586 h and 1188 h, respectively). Interestingly, we found that the rapid burn-in loss process^7,57^ of the PM6:PC_71_BM device has been effectively inhibited by the application of Ir/IrO_x_ nanoparticles, with an enhanced mean *T*_80_ of 328 h from 2.5 h in the ZnO-based device (Supplementary Fig. 23).

To understand the effect of electron-transporting materials on the stability of devices, device physics studies were further performed. Determined from TPC characterization shown in Supplementary Fig. 24, the charge extraction time *τ*_extra_ of aged devices with ZnO and Ir/IrO_x_ ETLs are 56.82 ns and 52.41 ns, which exhibits that a faster electron extraction process occurs in the aged Ir/IrO_x_-based device. In *P*_light_-*J*_sc_ dependent tests, the *α* amounts to 0.97 and 0.99, and the slopes of *P*_light_-*V*_oc_ curves are 1.13 *k*_B_T/q and 1.05 *k*_B_T/q for the aged ZnO and Ir/IrO_x_ devices, respectively (Supplementary Fig. 25). These analyses point to mitigated bimolecular charge recombination in the aged Ir/IrO_x_-based device. Besides, the bias-dependent EQE spectra provide information about the competition between charge extraction and charge recombination^58^. With the increasing forward bias, the value of *J*_sc_^cal^ decreases (Supplementary Fig. 26 and Supplementary Table 9), which indicates that the recombination is intensified upon reducing the internal electrical field. When the reverse bias reaches 0.8 V, the normalized *J*_sc_^cal^ with regard to *J*_sc_^cal^ at 0 V of fresh and aged devices with ZnO decreases to 48.19% and 36.06%, respectively. While, the values of fresh and aged devices with Ir/IrO_x_ ETL are 60.34% and 67.19%, which illustrates that the Ir/IrO_x_ can restrain the current loss due to recombination in both fresh and aged OSCs.

The above discussions on device physics studies confirm the inhibited charge recombination and accelerated charge extraction in the Ir/IrO_x_ devices after aging, which may be beneficial from the more stable BHJ morphology induced by the bottom Ir/IrO_x_ ETL. In the next section, the effect of Ir/IrO_x_ on the morphology evolution of BHJ films during aging will be discussed in detail.

### Morphology evolution of BHJ film

The complex morphology of BHJ films was deciphered hierarchically from molecular distribution and aggregation to phase separation. The molecular distribution was explored by XPS characterizations, for which a PM6:Y6 film with a thickness of 10 nm was used to mimic the bottom surface of the BHJ layers. The content of Nitrogen (${\omega }_{N}$) of the fresh film on Ir/IrO_x_ and ZnO is 3.62% and 3.30% (Supplementary Fig. 27 and Supplementary Table 10), which indicates that more Y6 molecules aggregate near the surface of Ir/IrO_x_ since N atom only exist in Y6 molecule. This behavior can be attributed to the larger *γ*_s_ and more pronounced HeD-SE property of the Ir/IrO_x_ ETL. After aging, the ${\omega }_{N}$ of aged BHJ film on Ir/IrO_x_ and ZnO is 3.48% and 2.77%, which indicates that the Ir/IrO_x_ stabilizes the molecular distribution during aging. This optimal and stable distribution of acceptor can assist to form a high-efficiency pathway for carrier transport and result in better PCE and stability^59^.

**Fig. 5:**
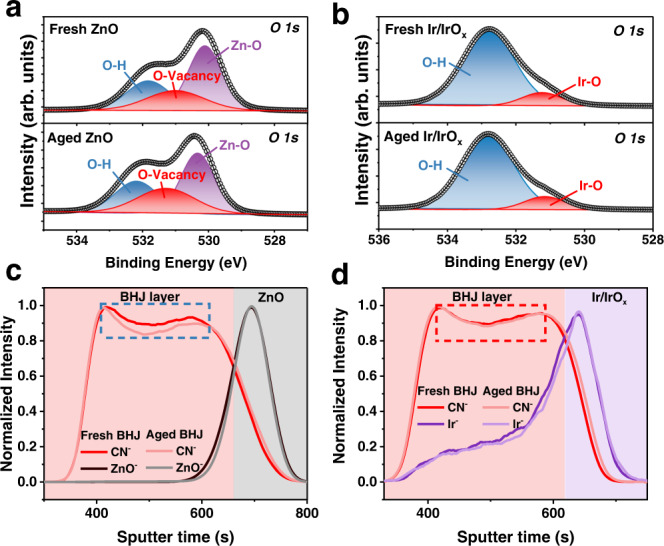
Molecular distribution. The XPS plots of core-level *O 1* *s* of fresh and aged ZnO (**a**) and Ir/IrO_x_ (**b**) films. The time-of-flight secondary ion mass spectrometry (TOF-SIMS) plots of PM6:Y6 BHJ films on ZnO (**c**) and Ir/IrO_x_ (**d**) ETLs.

To clarify why the molecular distribution became more stable, we further explored the change of the bottom ZnO and Ir/IrO_x_ with removing the BHJ films. Comparing with the *O 1* *s* core-level XPS spectrum of the fresh ZnO film, the ratio of O-vacancy increases in the aged ZnO film (Fig. 5a and Supplementary Table 11), which may link to the well-known photocatalysis problem and then impair the stability of OSCs. While, the O-vacancy signal is non-observable in both fresh and aged Ir/IrO_x_ films (Fig. 5b and Supplementary Table 12), which illustrates that the Ir/IrO_x_ nanoparticles are more stable than the ZnO ETL and able to inhibit the possible decomposition from photocatalysis. The time-of-flight secondary ion mass spectrometry (TOF-SIMS) characterization (Fig. 5c, d) was aimed to investigate the vertical distribution of Y6 in BHJ films on different ETLs. After aging, the content of the cyano group (CN, from Y6) decreases in the BHJ film on ZnO ETL, whereas the change in the BHJ film on Ir/IrO_x_ nanoparticles is negligible. This result further demonstrates the pronounced impact of Ir/IrO_x_ nanoparticles on the stability of the vertical distribution of BHJ films.

**Fig. 6:**
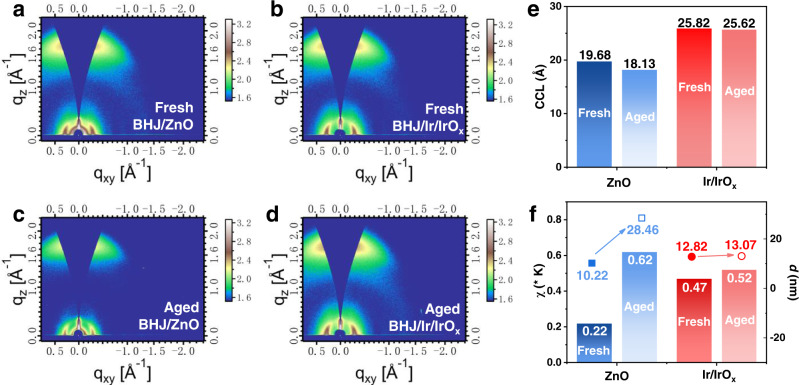
Morphology evolution of BHJ films. The two-dimensional (2D) grazing incidence wide-angle X-ray diffraction (GIWAXS) patterns of fresh (**a**) and aged (**b**) PM6:Y6 film on ZnO; fresh (**c**) and aged (**d**) PM6:Y6 film on Ir/IrO_x_. The comparison of coherence length (CCL) (**e**), Flory-Huggins parameters (*χ*), and domain size (*d*) (**f**) of fresh and aged BHJ films on ZnO and Ir/IrO_x_ electron-transporting materials. In **f**, the bars correspond to the *χ* parameters and the dots correspond to the *d* parameters extracted from the grazing incidence small angle X-ray scattering (GISAXS).

Grazing incidence wide-angle X-ray diffraction (GIWAXS) characterization (Fig. 6a–d) was performed to study the effect of Ir/IrO_x_ nanoparticles on molecular aggregation in BHJ films. The strong signal at *q* = 1.74 Å^−1^ in the out-of-plane (OOP) direction can be attributed to the π-π stacking of PM6 and Y6^60^. Based on the related cut-of-plane plots (Supplementary Fig. 28), the crystalline coherence length (CCL) of *π*-*π* stacking was calculated by the equation that CCL = 2πk/FWHM, where *k* equals 0.9, FWHM is the half-width of the diffraction peak. The ΔCCL was used to compare the CCL variation of fresh and aged BHJ films, which is defined by the equation that ΔCCL = (CCL_aged_−CCL_fresh_)/CCL_fresh_ × 100%. In Fig. 6e and Supplementary Table 13, the CCL of PM6:Y6 film on ZnO ETL decreases from 19.68 Å (fresh) to 18.13 Å (aged) (ΔCCL = −7.88%), while the CCL of fresh and aged BHJ films on Ir/IrO_x_ nanoparticles is 25.82 Å and 25.62 Å (ΔCCL = −0.77%), respectively. This result illustrates that the π-π stacking and the stability of the molecular packing of BHJs are enhanced by the Ir/IrO_x_ nanoparticles.

The Flory-Huggins parameter (*χ*) reflecting the miscibility of the donor-acceptor predicts the phase separation in the BHJ layer^61^. In Supplementary Fig. 29 and Supplementary Table 14, the parameter of *χ* was calculated by the equation described as *χ* = *K*${(\sqrt{{\gamma }_{{{\mbox{D}}}}}-\sqrt{{\gamma }_{{{\mbox{A}}}}})}^{2}$, where *γ*_D_ and *γ*_A_ are surface energies of the donor and acceptor and *K* is a constant parameter. The parameter Δ*χ* is defined to compare the *χ*-parameter variation of aged films with fresh films and its equation can be found in Supplementary Table 14. In Fig. 6f, the *χ* parameter of BHJ films on ZnO increases 182% from 0.22 *K* to 0.62 *K*. While, the *χ* parameter of BHJ films on Ir/IrO_x_ nanoparticles are 0.47 *K* (fresh) and 0.52 *K* (aged) with a much smaller Δ*χ* of 11%, which indicates that the phase separation of BHJ films on Ir/IrO_x_ is more stable than the film on ZnO ETL.

We also performed the grazing incidence small angle X-ray scattering (GISAXS) characterization to obtain the domain size (*d*) of phase separation in BHJ films. We compared the domain size variation (Δ*d*) of fresh and aged films, which expression can be found in Supplementary Table 15. In Fig. 6f, Supplementary Fig. 30 and Supplementary Table 14, the *d* parameters of fresh and aged BHJ films on ZnO are 10.22 nm and 28.46 nm, and the corresponding Δ*d* is 178%. While, the *d* of BHJ films on Ir/IrO_x_ only increases by 2% from 12.82 nm (fresh) to 13.07 nm (aged), which proves that the Ir/IrO_x_ stabilizes the phase separation of BHJ film and could further improve the lifetime of OSCs. Moreover, AFM characterizations also indicate that the BHJ film on Ir/IrO_x_ exhibits a more well-organized and stable surface morphology and fiber-like structure^62,63^ (Supplementary Figs. 31, 32). We consider that the Ir/IrO_x_ possesses a strong ability to make Y6 preferentially distribute to the modified ITO surface, stabilize the component distribution and morphology of BHJ films.

The above results of morphologic characterizations were summarized in Supplementary Fig. 33 to illustrate the morphology evolution of BHJ films on different ETLs before and after aging, described as follows: (i) In the fresh BHJ films, the higher γ_s_ and HeD-SE properties of Ir/IrO_x_ could lead to more Y6 molecule aggregate near to the interface between the Ir/IrO_x_ and BHJ film, which further results in the increase of π-π stacking and optimization of phase separation in BHJ, compared with the one on ZnO ETL. (ii) After aging, the morphology of BHJ on ZnO ETL is changed obviously, while it remains relatively stable on Ir/IrO_x_ ETL.

### Device stability under extreme conditions

**Fig. 7:**
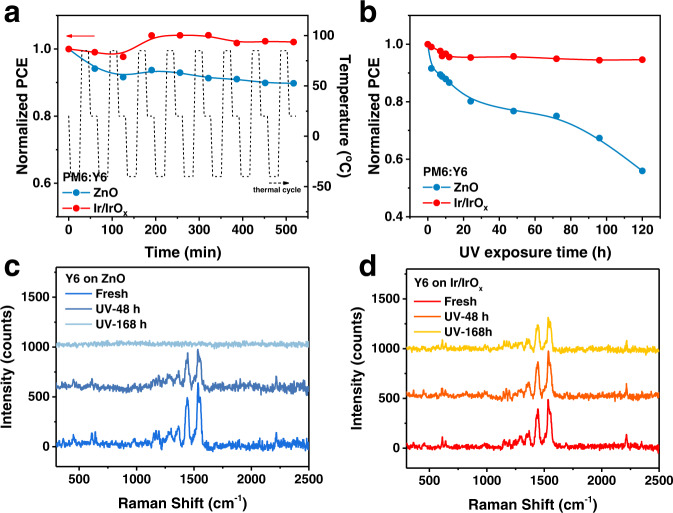
Device stability under extreme conditions. **a** The evolution plots of PCE with thermal-circulation time based on the champion PM6:Y6 devices with different ETLs. The thermal-circulation test was performed with the standard ISOS-T-3, in which the temperature range was from −40–85°C. **b** The evolution plots of PCE with ultraviolet (UV)-irradiation time based on the champion PM6:Y6 devices with different ETLs. The Raman spectra of Y6 films on ZnO (**c**) and Ir/IrO_x_ (**d**) during UV-irradiation aging.

Given that the Ir/IrO_x_-based OSCs exhibited excellent stability, we further explored the stability of devices under extreme conditions. In Fig. 7a, to explore the stability of OSCs under extreme temperature changes on Earth, we track the thermal-circulation stability of devices with the standard ISOS-T-3^7^. The PCE evolution of the ZnO-based device exhibits an obvious decline with increasing the number of cycles. After 9 cycles, the PCE of the best ZnO-based device decreases to 90% of the initial PCE, while the champion Ir/IrO_x_-based device even shows a higher value than the initial one (see the champion result in Fig. 5a and averaged results in Supplementary Fig. 34).

Moreover, the stability of OSCs exposed to continuous UV irradiation was also tracked. After 120 h exposure to ultraviolet (UV) light, the mean PCE, *V*_oc_, and FF of the ZnO-based device decreased to 53%, 90%, and 61% of respective initial values (Fig. 7b and Supplementary Fig. 35). While, all mean parameters of the device on Ir/IrO_x_ still maintain over 94% of the initial values and the champion device can maintain 95% of initial PCE. This is also the best *T*_95_ reported on non-fullerene OSCs under UV irradiation^64–68^. Raman spectra were applied to track the evolution of Y6 molecular structure on different ETLs during the UV-irradiation process. In Fig. 7c, d, the signal of carbon-carbon double bond (C=C, at 1536 cm^−1^) in Y6 molecular^69,70^ on ZnO decreases rapidly and disappears after 168 h of UV irradiation. Instead, the signal of the sample on Ir/IrO_x_ exhibits a more retarding decay tendency, which is similar to the one of the Y6 film on ITO substrate (Supplementary Fig. 36). It demonstrates that there is no obvious decay of Y6 induced by Ir/IrO_x_, and the Ir/IrO_x_ nanoparticles can efficiently improve the UV-irradiation stability of OSCs. Since the pronounced HeD-SE property benefits the formation of better acceptor distribution and the absence of photocatalysis ensures the stability of BHJ layer, the BHJ film on the Ir/IrO_x_ possesses more well-organized and stable morphology than the film on ZnO, which further inhibits the device degradation and extends the device lifetime. The results illustrate that the possibility of operating under extreme environments can be increased by applying Ir/IrO_x_ nanoparticles.

## Discussion

In conclusion, we demonstrate that the PCE and stability of OSCs can be enhanced simultaneously by a stable Ir/IrO_x_ ETL, which benefits from its suitable work function, the regulation of the optical field, the heterogeneous distribution of surface energy, and the absence of photocatalysis. Importantly, the champion devices with Ir/IrO_x_ exhibit superior long-term stabilities under shelf storing (*T*_80_ = 56,696 h vs. 12,075 h), thermal aging (*T*_70_ = 13,920 h vs. 2198 h), and MPP tracking (*T*_80_ = 1058 h vs. 586 h) when compared with the ZnO-based devices. It can be attributed to the stable morphology of photoactive layer resulting from the optimized molecular distribution of the donor and acceptor and the absence of photocatalysis in the Ir/IrO_x_-based devices, which helps to maintain the improved charge extraction and inhibited charge recombination in the aged devices. Moreover, the stable Ir/IrO_x_ can be utilized for OSCs operated in extreme conditions (including thermal circulation and UV irradiation) to improve the stability of devices and broaden the application scenarios of OSCs. This work provides a reliable and efficient electron-transporting material toward stable OSCs.

## Methods

### Materials

IrCl_3_·3H_2_O was purchased from Aladdin Reagents Co. Ltd. Ethylene glycol (≥99%), NaOH (≥96%), concentrated hydrochloric acid, and anhydrous ethanol was purchased from Sinopharm Chemical Reagents Co. Ltd. Chloroform (CF), chlorobenzene (CB) and methanol were purchased from Sigma-Aldrich and Acros, respectively. PM6, Y6 and PC_71_BM were purchased from Solarmer Material Inc. DIO, 1-chloronaphthalene (CN) and 2-methoxyethanol were purchased from TCI. PNDIT-F3N was purchased from eflex PV. Zn(Ac)_2_.(H_2_O)_2_ and MoO_x_ were purchased from Alfa Alser and Stream Chemical Inc., respectively. Ethanediamine was purchased from Acros.

### Synthesizing of Ir/IrO_x_ nanoparticles

We employed the mild colloid solution method to obtain the Ir/IrO_x_ nanoparticles. The detailed diagram of synthesizing route was exhibited in Supplementary Fig. 1 and detailed synthesis procedures were described below.

1. The 1.0 g IrCl_3_·3H_2_O precursor was dissolved in 150 ml ethylene glycol under stirring and the value of pH was adjusted to 10 by adding 0.25 mol/L NaOH/glycol solution.
2. Then, the blend solution was heated at 160 °C under Ar atmosphere for 3 hours.
3. After heating for 3 hours, the brown Ir/IrO_x_ colloid solution (0.39 wt % ≈ 4.5 mg/ml) can be obtained after cooling down to room temperature.

### Device fabrication

Firstly, ITO-coated glass substrates were cleaned with distilled water, acetone, and isopropanol (IPA) in an ultrasonic bath followed by ultraviolet-ozone treatment for 15 minutes. A solution of ZnO precursor was spin-coated onto the ITO surface at 3000 rpm and thermally annealed at 200°C for 30 minutes. In the process of optimization, the solution of Ir/IrO_x_ was spin-coated onto the ITO substrate with different speeds and then was thermally annealed at different temperatures and time. Then, the active layer solution was spin-coated with optimized speed. After the spin-coating process, the PM6:PC_71_BM film did not experience any annealing process, while PM6:Y6 and PM6:Y6:PC_71_BM were further thermally annealed at 110°C for 10 minutes. After this step, MoO_x_ (8 nm) was thermally evaporated under a high vacuum (ca. 3 × 10^−4 ^Pa) as a hole-transporting layer in OSCs. Finally, Al (80 nm) was thermally evaporated under a high vacuum (ca. 3 × 10^−4 ^Pa). The electron-only devices were fabricated based on the following structure: ITO/ZnO(or Ir/IrO_x_)/PM6:Y6/PNDIT-F3N/Ag. In the fabrication process of electron-only devices, the ZnO and Ir/IrO_x_ were spin-coated under the same conditions as the organic solar cells.

### TEM characterization

The transmission electron microscope (TEM) was applied to obtain the cross-sectional view patterns of the Ir/IrO_x_ film on ITO substrate. The samples for the cross-sectional TEM were prepared by the focused ion beam (FIB). The special aberration-corrected transmission electron microscope (AC-TEM) was performed on the 300 kV Thermo Fisher Spectra 300. For the AC-TEM characterization, the colloidal solution of Ir/IrO_x_ nanoparticles was diluted to 1% of the initial concentration with methanol. Then, the solution was dripped onto the copper mesh using a pipette gun.

### XPS and UPS characterizations

XPS and UPS were tested by multifunctional photoelectronic energy spectrometer ESCALAB250XI, Thermo Fisher Scientific. To fabricate the sample of XPS and UPS, the colloidal solution of Ir/IrO_x_ nanoparticles was spin-coated on the ITO substrate using the same conditions as the device fabrication.

### AFM and PFQNM characterizations

AFM morphology images and PFQNM adhesion mappings were characterized by Bruker Multimode 8HR. A tip coating Pt/Ir with a nominal spring constant of 2.9 N/m and a tip radius of 25 nm was used for morphology and adhesion measurements.

### *J-V* characteristics and EQE

The *J*−*V* characteristics were performed by the solar simulator (SS-F5-3A, Enlitech) along with AM 1.5 G spectra which intensity was calibrated by the certified standard silicon solar cell (SRC-2020, Enlitech) at 100 mW/cm^2^. EQE spectra were measured by a Solar Cell Spectral Response Measurement System QE-R3011 (Enlitech, Taiwan).

### Device attenuation test

To perform the test of shelf stability, devices were stored in an N_2_-filled glovebox at room temperature. In the test of thermal aging, devices were placed on a high-precision hotplate heating at 60°C in an N_2_-filled glovebox. The operating stability of devices was tested by a Photovoltaic Performance Decay Testing System (D&R Instruments) under illumination with the intensity equivalent to 1 sun (100 mW/cm^2^) at the maximum power point (MPP). To study the aged device mechanism and morphologic evolution, BHJ films spin-coated on different electron-transporting materials were placed under illumination (100 mW/cm^2^) and heated at 85°C to accelerate the aging process. For the stability of thermal circulation, devices were placed in a vacuum chamber and the temperature was set according to the standard ISOS-T-3. To observe the aging process of UV-irradiation stability, devices or Y6 films were exposed to 365 nm UV light.

### Reporting summary

Further information on research design is available in the Nature Portfolio Reporting Summary linked to this article.

## Supplementary information

Supplementary Information

Solar Cells Reporting Summary

## Data Availability

The data that support the findings of this study are available from the corresponding authors.
