# Supplementary material for: Lifetime over 10000 hours for organic solar cells with Ir/IrOx electron-transporting layer
Source: Nat Commun. 2023 Mar 4;14:1241. doi: 10.1038/s41467-023-36937-8 (PMC9985642; doi:10.1038/s41467-023-36937-8)
Supplement: Supplementary file 1 — Supplementary Information [file 41467_2023_36937_MOESM1_ESM.pdf]

Supplementary Information for

Lifetime over 10000 Hours for Organic Solar Cells with Ir/IrO_x Electron-transporting layer

Yanxun Li[1,2], Bo Huang[1], Xuning Zhang[3], Jianwei Ding[4], Yingyu Zhang[1,2], Linge Xiao[1,2], Boxin Wang[1,2], Qian Cheng[1,2], Gaosheng Huang[1,2], Hong Zhang[1], Yingguo Yang[5], Xiaoying Qi[4], Qiang Zheng[4], Yuan Zhang[3], Xiaohui Qiu[4], Minghui Liang[1], Huiqiong Zhou[1,2*]

[1] CAS Key Laboratory of Nanosystem and Hierarchical Fabrication, National Center for Nanoscience and Technology, Beijing 100190, P. R. China
[2] Center of Materials Science and Optoelectronics Engineering, University of Chinese Academy of Sciences, Beijing 100049, P. R. China
[3] School of Chemistry, Beijing Advanced Innovation Center for Biomedical Engineering, Beihang University, Beijing 100191, P. R. China
[4] CAS Key Laboratory of Standardization and Measurement for Nanotechnology, National Center for Nanoscience and Technology, Beijing 100190, P. R. China
[5] Shanghai Synchrotron Radiation Facility (SSRF), Zhangjiang Lab, Shanghai Advanced Research Institute, Chinese Academy of Sciences, Shanghai 201204, China.
* Corresponding authors, Email: zhouhq@nanoctr.cn
These authors contributed equally: Yanxun Li, Bo Huang.

Keywords: iridium nanoparticle, electron-transporting layer, organic solar cells, stability, morphology

Contents

Supplementary Methods 4

Solution preparation 4

Characterizations 4

Supplementary Figures and Tables 6

Section 1. Characterizations of Ir/IrO_x nanoparticles in colloid solution 6

Supplementary Fig. 1 6

Supplementary Fig. 2 6

Supplementary Fig. 3 6

Section 2. Characterizations of Ir/IrO_x nanoparticles and ZnO deposited on ITO 7

Supplementary Fig. 4 7

Supplementary Fig. 5 7

Supplementary Fig. 6 8

Supplementary Fig. 7 8

Supplementary Fig. 8 8

Supplementary Fig. 9 9

Supplementary Table 1 9

Section 3. The optimization of Ir/IrO_x-based device performance 10

Supplementary Fig. 10.. 10

Supplementary Table 2 10

Supplementary Table 3 11

Supplementary Table 4 11

Supplementary Fig. 11.. 12

Supplementary Table 5 13

Supplementary Fig. 12.. 13

Supplementary Table 6 14

Section 4. The optical properties of Ir/IrO_x and ZnO 15

Supplementary Fig. 13.. 15

Supplementary Fig. 14.. 15

Supplementary Fig. 15.. 15

Section 5. The device mechanism of fresh devices on ZnO and Ir/IrO_x 16

Supplementary Fig. 16.. 16

Supplementary Fig. 17.. 16

Section 6. The long-term stability 17

Supplementary Fig. 18.. 17

Supplementary Fig. 19.. 17

Supplementary Table 7 17

Supplementary Table 8 18
Supplementary Fig. 20.......... 18
Supplementary Fig. 21.......... 19
Supplementary Fig. 22.......... 19
Supplementary Fig. 23.......... 20
Supplementary Fig. 24.......... 20
Supplementary Fig. 25.......... 20
Supplementary Fig. 26.......... 21
Supplementary Table 9 21
Section 7. The morphology evolution of BHJ films on different interlayers 22
Supplementary Fig. 27.......... 22
Supplementary Table 10 22
Supplementary Table 11.......... 23
Supplementary Table 12 23
Supplementary Fig. 28.......... 24
Supplementary Table 13 24
Supplementary Fig. 29.......... 25
Supplementary Table 14 25
Supplementary Fig. 30.......... 26
Supplementary Table 15 26
Supplementary Fig. 31.......... 27
Supplementary Fig. 32.......... 27
Supplementary Fig. 33.......... 27
Section 8. Device stability under extreme conditions 28
Supplementary Fig. 34.......... 28
Supplementary Fig. 35.......... 28
Supplementary Fig. 36.......... 29
Supplementary References.......... 30

Supplementary Methods

Solution preparation

100 mg $Zn(Ac)_2.(H_2O)_2$ has been dissolved in 1 mL 2-methoxyethanol and 30 μL ethanediamine has been added in solution. Then, the composite solution has been stirred in room temperature for 24 hours. Ir/IrO_x colloid solution was diluted to different concentrations for device optimization by adding glycol solution with different volume and these solutions were stirred in room temperature for 2 hours. 0.5 mg PNDIT-F3N was dissolved in 1 mL methanol and 10 μL acetic acid was added as an additive.

The PM6:Y6 (1:1.2, wt/wt) and PM6:Y6:$PC_{71}BM$ (1:1:0.2) mixtures were dissolved in chloroform with a total concentration of 16 mg/mL, respectively. The active layer solution was stirred at room temperature for at least 2 hours and 0.5 % CN (CN: CB, v/v) was added 30 minutes prior to the spin-coating. PM6:$PC_{71}BM$ (1:1, wt/wt) mixture are dissolved in CB with donor concentration of 10 mg/ml. The active layer solution was stirred at room temperature for 12 hours and 0.5% DIO (DIO:CB, v/v) was added in active layer solution.

Characterizations

Contact angle was measured by using an automatic contact angle measuring instrument (DSA-100). GIWAXS patterns were examined by WZX-SAXS/WAXS, Xenocs. GISAXS characterization was performed by BL16B1 beamline in SSRF.

The *J*−*V* characteristics was performed by the solar simulator (SS-F5-3A, Enlitech) along with AM 1.5G spectra which intensity was calibrated by the certified standard silicon solar cell (SRC-2020, Enlitech) at 100 mW/cm^2. External quantum efficiency (EQE) spectra were measured by a Solar Cell Spectral Response Measurement System QE-R3011 (Enlitech, Taiwan).

The electron mobility was calculated by SCLC method using the formula described as $J=\frac{9}{8}\varepsilon_0\varepsilon_r\mu\frac{V^2}{d^3}$ where *J* is the current density, μ is the charge carrier mobility, are the permittivity of the vacuum and the relative permittivity of the material, *d* is the thickness of the blend film and *V* is the effective voltage. Transient photovoltage (TPV) and transient photocurrent (TPC) were tested by a customized transient measurement

systems (Physike Technology Co., Ltd) with a pulsed semiconductor laser (Coherent, Inc.). The electrochemical impedance spectroscopy was performed by the CHI660 electrochemical workstation (CH Instrument, Inc.) and devices were tested under solar illumination (AM 1.5G). The Raman spectra was obtained by the Renishaw in Via plus.

Supplementary Figures and Tables

Section 1. Characterizations of Ir/IrO$_x$ nanoparticles in colloid solution

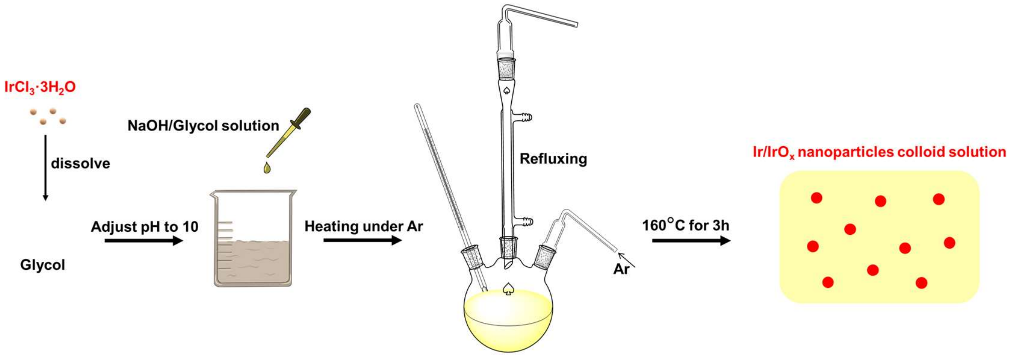

Supplementary Fig. 1. **Synthesizing route of Ir/IrO$_x$.** The synthesizing route of Ir/IrO$_x$ nanoparticles in colloid solution and the diagram of reaction equipment.

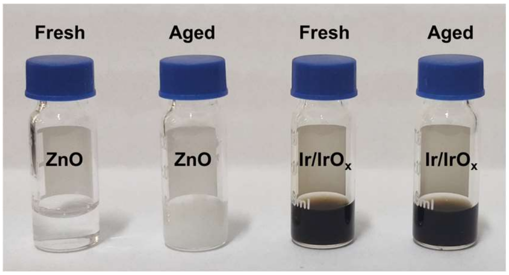

Supplementary Fig. 2. **Solution photographs.** Photographs of fresh and aged (storing in air for 2 years) ZnO- precursor solution and Ir/IrO$_x$ colloid solution.

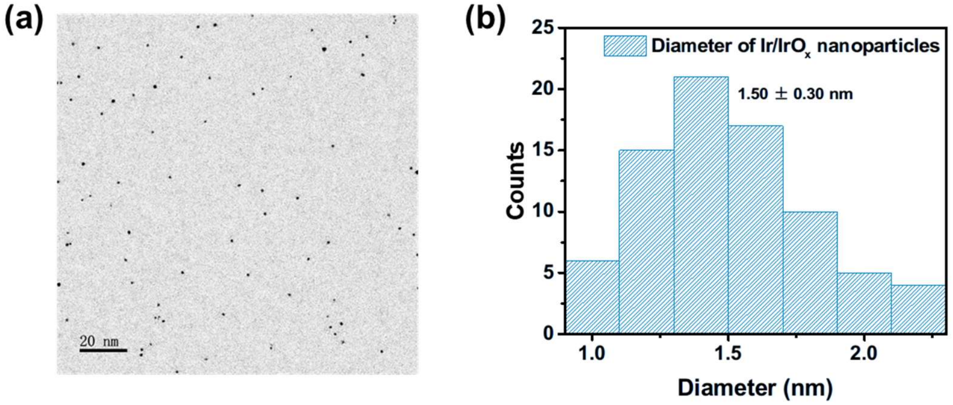

Supplementary Fig. 3. **The AC-TEM characterization.** (a) The AC-TEM pattern of Ir/IrO$_x$ nanoparticles deposited on the cooper mesh and (b) the corresponded statistics of diameter distribution.

Section 2. Characterizations of Ir/IrO_x nanoparticles and ZnO deposited on ITO

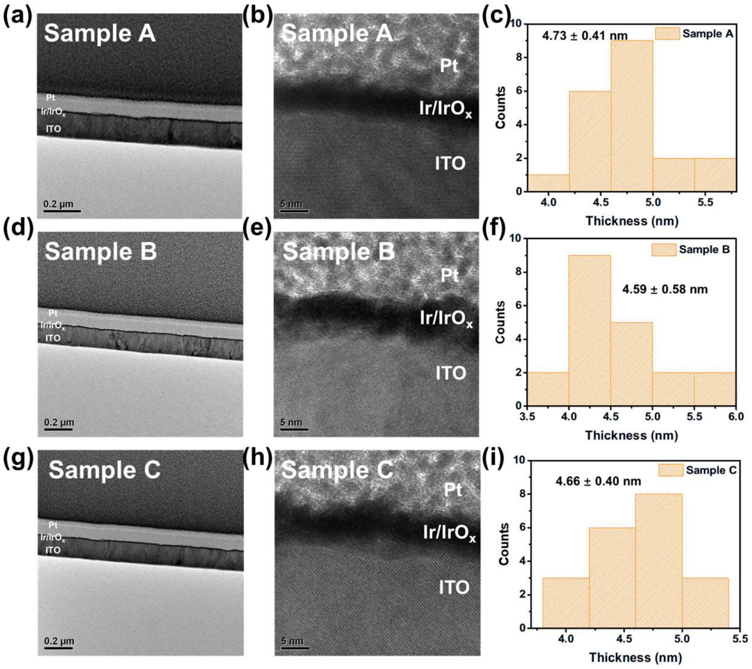

Supplementary Fig. 4. **The cross-section TEM characterization.** The cross-sectional TEM patterns of three Ir/IrO_x-film samples (a, b) Sample A, (d, e) B and (g, h) C. The TEM samples (A, B and C) were cut from different positions on $ITO/Ir/IrO_x$ film by focused ion beam (FIB) . (c, f, i) The extracted statistics of thickness of the Ir/IrO_x film. Each sample were selected 20 places from the high-resolution TEM images to measure the thickness and further calculate the mean thickness of Ir/IrO_x film.

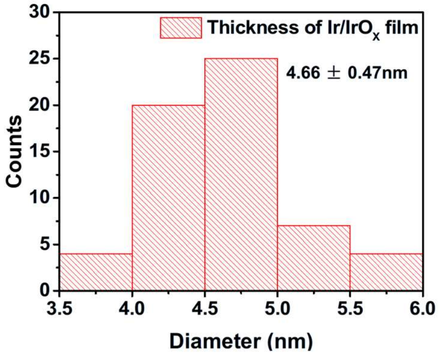

Supplementary Fig. 5. **The statistics of Ir/IrO_x-film thickness.** The statistics of thickness extracted from the three cross-sectional TEM patterns. The mean thickness was calculated based on the 60 places from above three high-resolution TEM patterns.

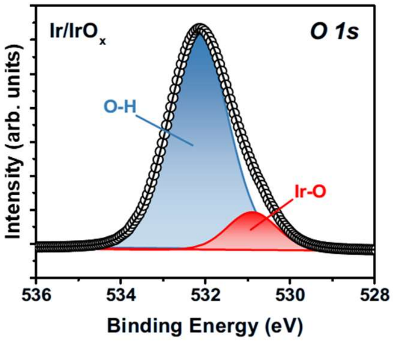

Supplementary Fig. 6. The XPS characterization of Ir/IrO$_x$. The XPS plot of *O 1s* in Ir/IrO$_x$.

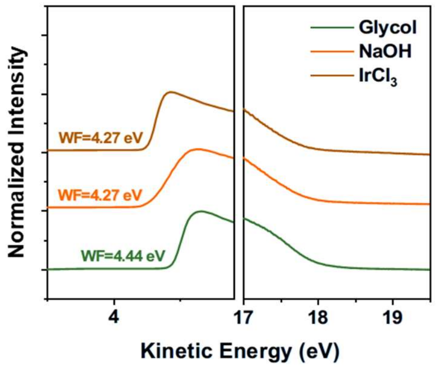

Supplementary Fig. 7. The UPS characterization. The UPS characterizations of ITO modified by glycol (green line), NaOH in glycol (pH=10, orange line) and $IrCl_3$ in glycol (NaOH, pH=10, brown line), respectively.

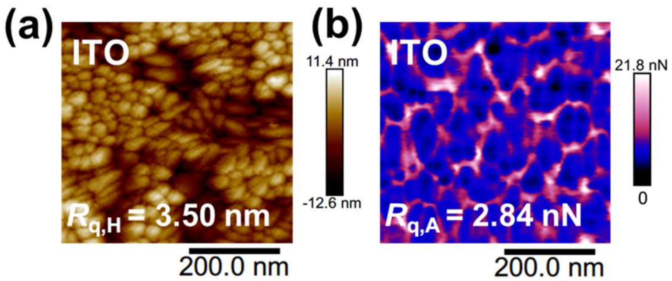

Supplementary Fig. 8. The AFM characterization of ITO. (a) AFM topography and (b) adhesion mappings of bare ITO.

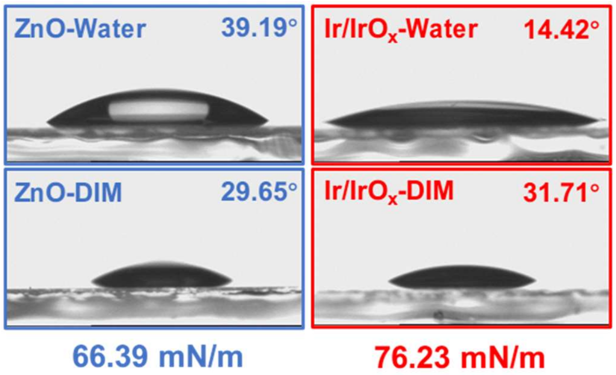

Supplementary Fig. 9. The contact angle and surface energy. The contact angle photographs of ZnO and Ir/IrO_x.

Supplementary Table 1. The statistics of interfacial parameters. The summary of root-mean-square roughness of height images ($R_{q,H}$) and adhesion images ($R_{q,A}$) in PFQNM characterization and the summary of surface energy in contact angle measurement.

Materials	$R_{q,H}$ (nm)	$R_{q,A}$ (nN)	γ_s (mN/m)
ITO	3.50	2.84	-
ZnO	2.02	1.34	66.39
Ir/IrO_x	3.09	5.92	76.23

Section 3. The optimization of Ir/IrO_x-based device performance

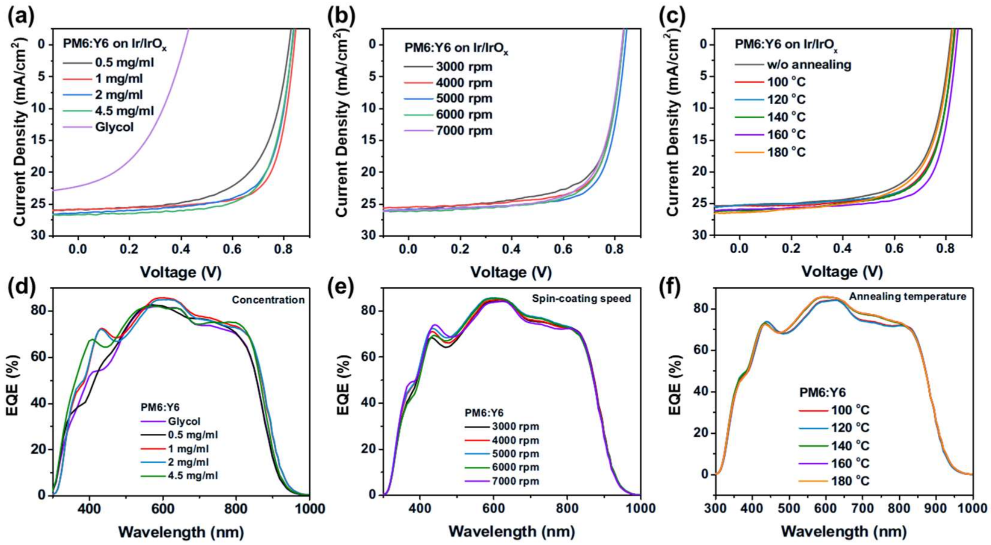

Supplementary Fig. 10. Device performance of devices during optimization. *J-V* characteristics and EQE curves of PM6:Y6 devices on different electron transporting materials with (a, d) different concentration, (b, e) various spin-coating speed and (c, f) different annealing temperature.

Supplementary Table 2. Device parameters of devices during Ir/IrO_x-concentration optimization. The summary of parameters for devices processed on Ir/IrO_x films, made from different Ir/IrO_x-concentrations. The spin-coating speed and the annealing temperature were kept at 5000 rpm and 160 °C, respectively.

Concentration (mg/ml)	V_{oc} (V)	J_{sc}[a] (mA/cm^2)	J_{sc} cal b (mA/cm^2)	FF (%)	PCE (%)
0 (glycol)	0.42	22.26	23.04	43.21	3.99
0.5	0.82	25.89	23.22	63.54	13.49
1	0.84	25.81	25.19	73.29	15.89
2	0.83	26.69	24.48	70.68	15.66
4.5	0.83	26.44	24.27	70.02	15.37

[a] Short-circuit (J_{sc}) is obtained from *J-V* characteristics.

[b] The integral current density of the external quantum efficiency (EQE) characterization.

Supplementary Table 3. Device parameters of devices during spin-coating-speed optimization. The summary of parameters for devices processed on Ir/IrO_x films, made with different spin-coating speed for Ir/IrO_x-deposition. During optimization of spin-coating speed, the concentration of Ir/IrO_x solution was 1 mg/ml and the annealing temperature was 160 °C.

Spin-coating speed (rpm)	V_{oc} (V)	J_{sc} [a] (mA/cm^2)	J_{sc} cal [b] (mA/cm^2)	FF (%)	PCE (%)
3000	0.83	25.80	24.24	67.28	14.46
4000	0.82	25.51	24.37	72.52	15.17
5000	0.84	25.81	25.19	73.29	15.89
6000	0.83	26.15	24.43	70.67	15.34
7000	0.82	25.99	24.24	70.20	14.96

[a] Short-circuit (J_{sc}) is obtained from *J-V* characteristics.

[b] The integral current density of the external quantum efficiency (EQE) characterization.

Supplementary Table 4. Device parameters of devices during annealing temperature optimization. The summary of parameters for devices processed on Ir/IrO_x films, made with different annealing temperature (T_a). When optimizing the annealing temperature, the Ir/IrO_x concentration was 1 mg/ml and all devices applied a spin-coating speed of 5000 rpm.

T_a (°C)	V_{oc} (V)	J_{sc}[a] (mA/cm^2)	J_{sc} cal [b] (mA/cm^2)	FF (%)	PCE (%)
w/o	0.82	25.28	-	66.42	13.76
100	0.83	25.21	24.16	69.98	14.64
120	0.83	25.18	24.09	70.97	14.83
140	0.83	26.07	24.66	68.95	14.92
160	0.84	25.81	25.19	73.29	15.89
180	0.82	26.40	24.71	65.07	14.08

[a] Short-circuit (J_{sc}) is obtained from *J-V* characteristics.

[b] The integral current density of the external quantum efficiency (EQE) characterization.

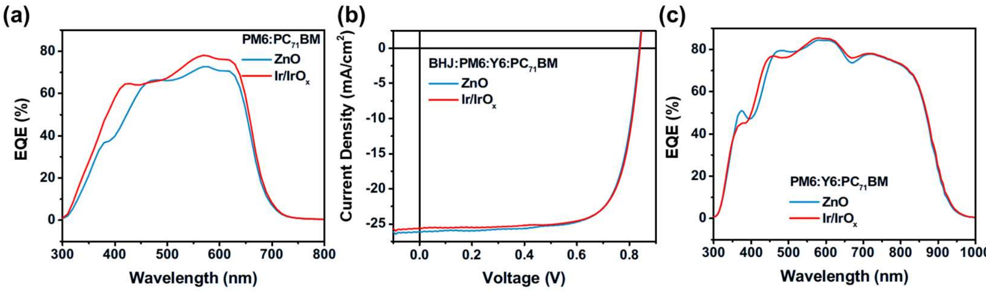

Supplementary Fig. 11. **Device performance of PM6:PC$_{71}$BM and PM6:Y6:PC$_{71}$BM devices.** (a) The EQE curves of PM6:PC$_{71}$BM devices on ZnO and Ir/IrO$_x$. The (b) *J-V* characteristics and (c) EQE curves of PM6:Y6:PC$_{71}$BM OSCs on different electron transporting materials.

Supplementary Table 5. Device parameters of optimal devices. The statistic of mean parameters for devices with different active layers.

BHJ	Electron transporting materials	V_{oc} [c] (V)	J_{sc} [a, c] (mA/cm^2)	J_{sc}^{cal} [b] (mA/cm^2)	FF [c] (%)	PCE [c] (%)
PM6:Y6	ZnO	0.83 ± 0.01 (0.84)	25.48 ± 0.35 (25.34)	24.63	71.98 ± 1.21 (73.21)	15.24 ± 0.22 (15.58)
	Ir/IrO_x	0.83 ± 0.01 (0.84)	25.98 ± 0.32 (25.81)	25.19	72.32 ± 0.62 (73.29)	15.73 ± 0.10 (15.89)
PM6: $PC_{71}BM$	ZnO	0.91 ± 0.01 (0.92)	12.68 ± 0.11 (12.95)	11.55	61.97 ± 0.22 (62.06)	7.12 ± 0.10 (7.38)
	Ir/IrO_x	0.92 ± 0.01 (0.92)	13.94 ± 0.26 (13.70)	12.59	63.73 ± 0.83 (64.50)	8.11 ± 0.03 (8.13)
PM6:Y6: $PC_{71}BM$	ZnO	0.83 ± 0.01 (0.84)	25.91 ± 0.18 (26.07)	24.41	71.63 ± 0.81 (72.92)	15.74 ± 0.15 (15.95)
	Ir/IrO_x	0.83 ± 0.01 (0.84)	25.80 ± 0.25 (25.58)	24.71	73.36 ± 0.86 (75.35)	16.06 ± 0.09 (16.19)

[a] Short-circuit (J_{sc}) is obtained from *J*-*V* characteristics.

[b] The integral current density of the external quantum efficiency (EQE) characterization.

[c] The average values and deviations were calculated based on over 8 devices.

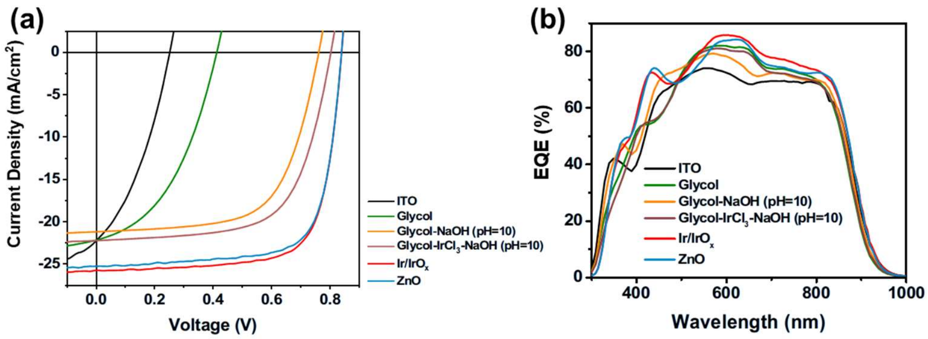

Supplementary Fig. 12. Device performance of devices with different ETLs. (a) *J*-*V* characteristics and (b) EQE curves of PM6:Y6 devices on different substrates including bare ITO, ITO with glycol, ITO with glycol-NaOH (pH=10), ITO with glycol-$IrCl_3$-NaOH (pH=10), ITO with ZnO and ITO with Ir/IrO_x nanoparticles.

Supplementary Table 6. Device parameters. Summary of device parameters of PM6:Y6 cells prepared with different substrates including bare ITO, ITO with glycol, ITO with glycol-NaOH (pH = 10), ITO with glycol-$IrCl_3$-NaOH (pH = 10), ITO with ZnO and ITO with Ir/IrO_x nanoparticles.

Substrate	V_{oc}[a] (V)	J_{sc}[a,b] (mA/cm²)	J_{sc} cal c (mA/cm²)	FF[a] (%)	PCE[a] (%)
ITO	0.20±0.04 (0.25)	19.71±2.39 (22.00)	22.09	35.47±1.46 (36.85)	1.42±0.50 (2.03)
with glycol	0.37±0.04 (0.42)	21.89±0.93 (22.26)	23.04	42.57±2.47 (43.21)	3.45±0.44 (3.99)
with glycol-NaOH (pH = 10)	0.68±0.06 (0.73)	23.21±1.17 (23.09)	23.07	59.69±4.26 (65.17)	9.46±1.05 (11.02)
with glycol-$IrCl_3$-NaOH (pH = 10)	0.78±0.02 (0.81)	21.71±0.96 (22.32)	22.79	60.70±3.54 (64.74)	10.38±0.89 (11.70)
with Ir/IrO_x	0.83 ± 0.01 (0.84)	25.98 ± 0.32 (25.81)	25.19	72.32 ± 0.62 (73.29)	15.73 ± 0.10 (15.89)
with ZnO	0.83 ± 0.01 (0.84)	25.48 ± 0.35 (25.34)	24.63	71.98 ± 1.21 (73.21)	15.24 ± 0.22 (15.58)

[a] The average values and deviations were calculated based on over 8 devices.

[b] Short-circuit (J_{sc}) is obtained from *J-V* characteristics.

[c] The integral current density of the external quantum efficiency (EQE) characterization.

Section 4. The optical properties of Ir/IrO$_x$ and ZnO

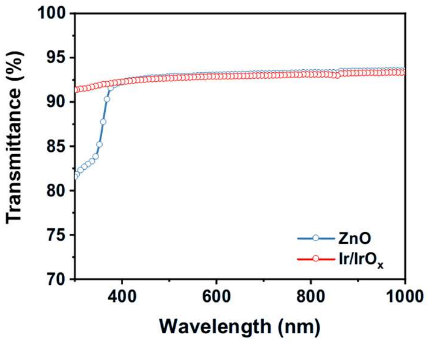

Supplementary Fig. 13. Transmittance spectra of ETLs. The transmittance spectra of ZnO and Ir/IrO$_x$.

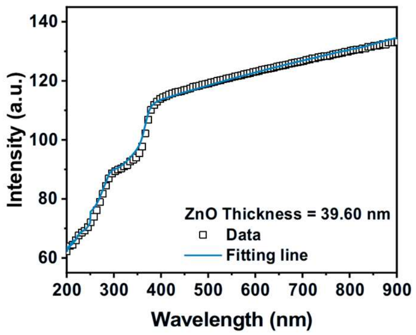

Supplementary Fig. 14. The ellipsometry data. Fitting curve of ellipsometry data corresponding to ZnO film. The thickness of ZnO thin films obtained by ellipsometry-data fitting is 39.6 nm.

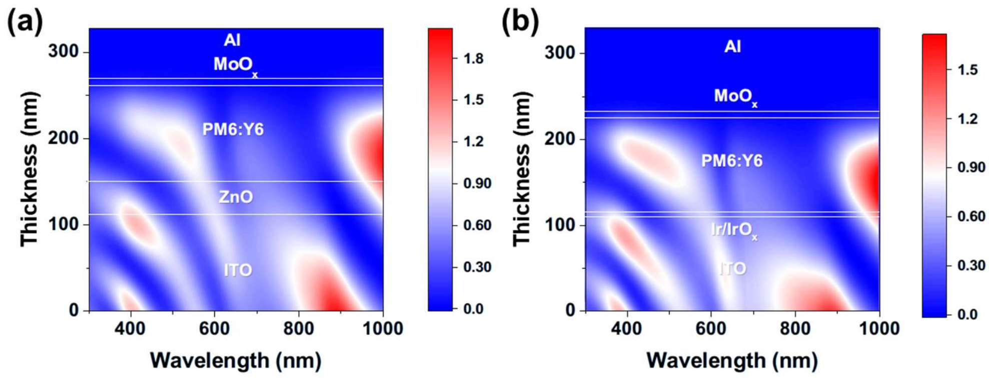

Supplementary Fig. 15. The optical simulations. Optical simulations for the optical field intensity $|E|^2$ profiles in the studied (a) ZnO and (b) Ir/IrO$_x$ -based OPV devices. Device structure used in the optical simulation are ITO/ZnO/PM6:Y6/MoO$_x$/Al and ITO/ Ir/IrO$_x$/PM6:Y6/MoO$_x$/Al, respectively.

Section 5. The device mechanism of fresh devices on ZnO and Ir/IrOx

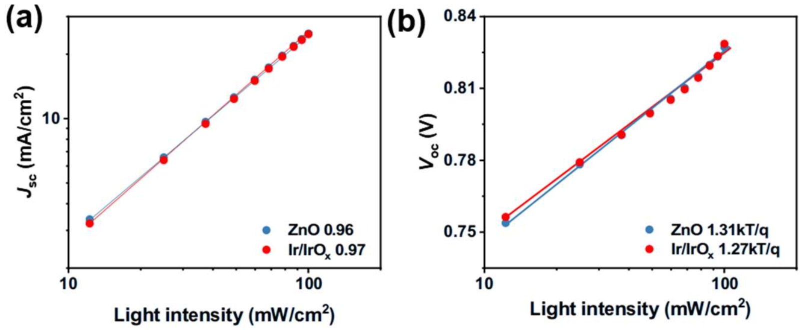

Supplementary Fig. 16. Light-intensity dependent characterizations. Light-intensity dependent curves of (a) J_{sc} and (b) V_{oc} for fresh PM6:Y6 devices on different electron transporting materials.

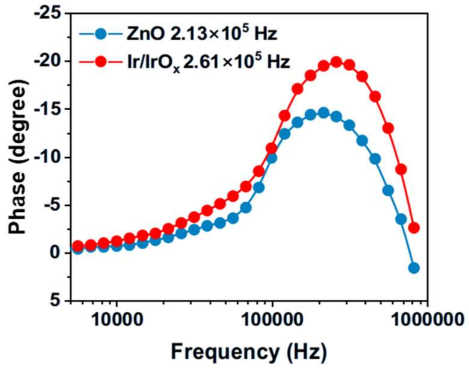

Supplementary Fig. 17. Bode plots. The Bode phase plots of PM6:Y6 devices on ZnO and Ir/IrO$_x$, which are extracted from the electrochemical impedance spectroscopy (EIS).

Section 6. The long-term stability

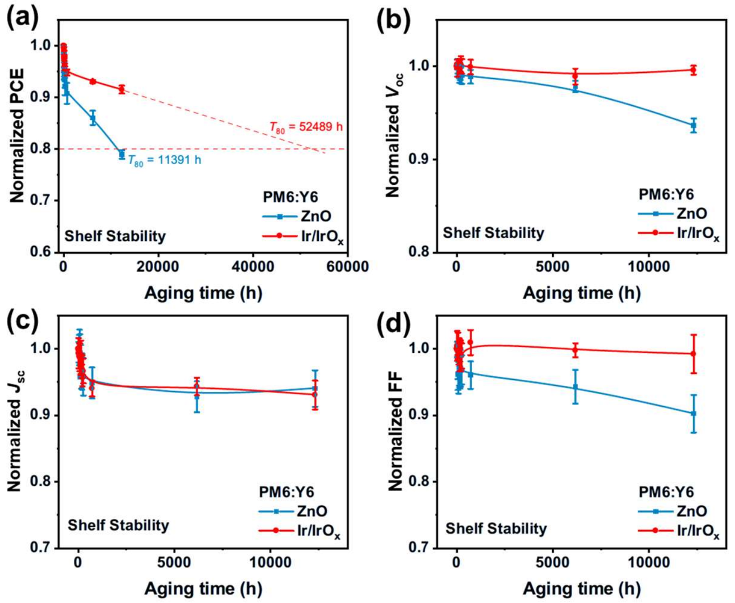

Supplementary Fig. 18. Attenuation of PM6:Y6-based device parameters under shelf storing. The evolution plots of (a) PCE and (b) V_{oc}, (c) J_{sc} and (d) FF of PM6:Y6 devices under shelf storing. The error bar is the standard deviation and it is calculated based on six devices for each interfacial material.

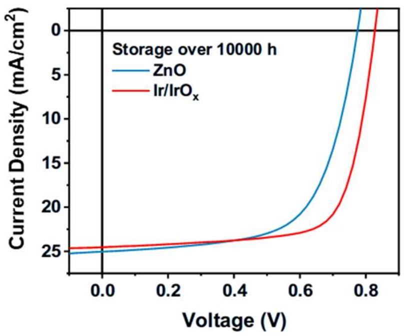

Supplementary Fig. 19. Device performance of aged devices. *J-V* characteristics of PM6:Y6 devices on different electron transporting materials after storing in N_2-filled glovebox for 10000 h.

Supplementary Table 7. Device parameters of aged devices. The summary of parameters for champion devices on ZnO and Ir/IrO$_x$ after 10000 h storage in N_2-filled glovebox.

HTL	V_{oc} (V)	J_{sc} (mA/cm^2)	FF (%)	PCE (%)
ZnO	0.77	25.04	64.32	12.42
Ir/IrO$_x$	0.83	24.54	72.34	14.67

Supplementary Table 8. The statistic of mean lifetime. The statistic of average T_{80} and T_{70} extracted from the stability test of devices under different aging conditions.

	Aging condition	T_{80} (h)	T_{70} (h)
ZnO	Shelf stability [a]	11391	-
	Thermal aging [b]	1320	2240
	MPP tracking [c]	580	1425[d]
Ir/IrOx	Shelf stability [a]	52489 [d]	-
	Thermal aging [b]	2495	10248
	MPP tracking [c]	1048	2005 [d]

[a] Average value is based on the parameters of six devices whose BHJ are PM6:Y6.

[b] Average value is based on the parameters of five devices whose BHJ are PM6:Y6.

[c] Average value is based on the parameters of three devices whose BHJ is PM6:Y6:$PC_{71}BM$.

[d] This lifetime was obtained by fitting the average PCE evolution plot of devices.

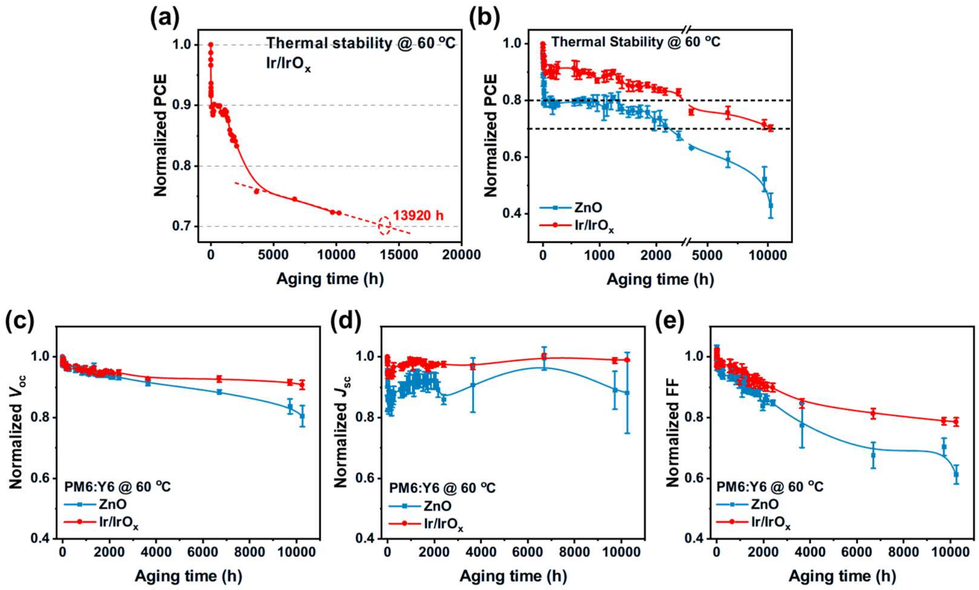

Supplementary Fig. 20. Attenuation of PM6:Y6-based device parameters under thermal aging. The evolution plots of (a, b) PCE and (c) V_{oc}, (d) J_{sc} and (e) FF of PM6:Y6 devices heating at 60℃. The error bar is the standard deviation and it is calculated based on five devices for each interfacial material.

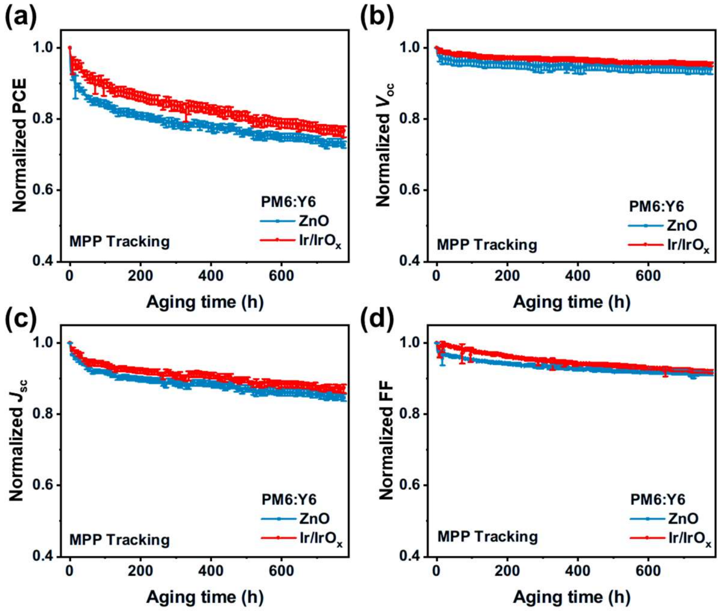

Supplementary Fig. 21. **Attenuation of PM6:Y6-based device parameters under MPP tracking.** The evolution plots of (a) PCE, (b) V_{oc}, (c) J_{sc} and (d) FF of PM6:Y6 devices tracking at MPP under continuous illumination which intensity was equivalent to 100 mW/cm^2. The error bar is the standard deviation and it is calculated based on three devices for each interfacial material.

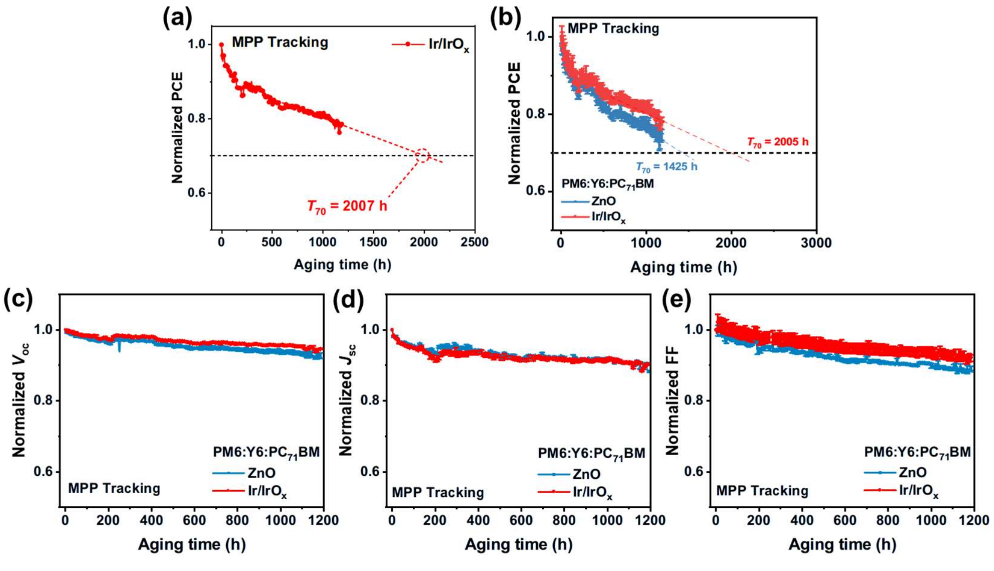

Supplementary Fig. 22. Attenuation of PM6:Y6:PC$_{71}$BM-based device parameters under MPP tracking. The evolution plots of (a, b) PCE, (c) V_{oc}, (d) J_{sc} and (e) FF of PM6:Y6:PC$_{71}$BM devices tracking at MPP under continuous illumination which intensity was equivalent to 100 mW/cm^2. The error bar is the standard deviation and it is calculated based on three devices for each interfacial material.

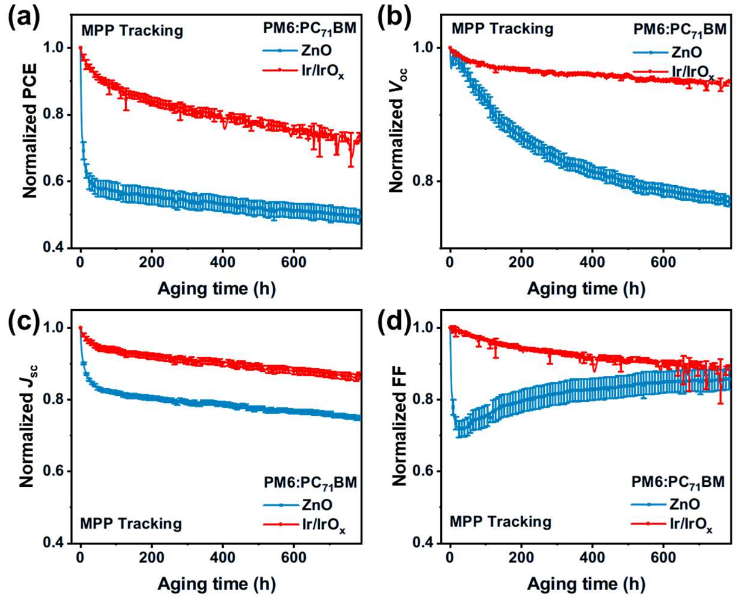

Supplementary Fig. 23. **Attenuation of PM6:PC_{71}BM-based device parameters under MPP tracking.** The evolution plots of (a) PCE, (b) V_{oc}, (c) J_{sc} and (d) FF of PM6:PC_{71}BM devices tracking at MPP under continuous illumination which intensity was equivalent to 100 mW/cm^2. The error bar is the standard deviation and it is calculated based on three devices for each interfacial material.

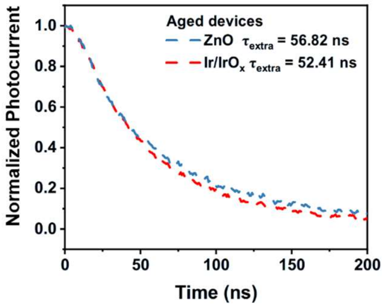

Supplementary Fig. 24. TPC characterizations of aged devices. TPC characterization of aged PM6:Y6 devies on ZnO and Ir/IrO_x.

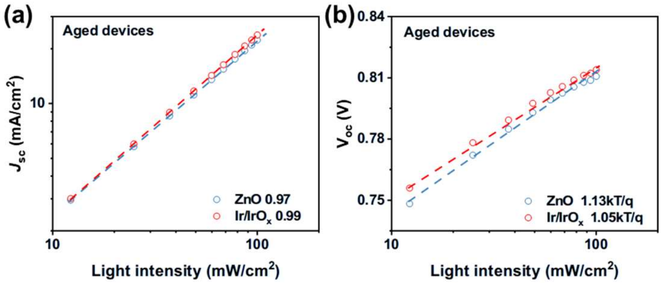

Supplementary Fig. 25. Light-intensity dependent characterizations of aged devices. Light-intensity dependent curves of (a) J_{sc} and (b) V_{oc} for aged PM6:Y6 devices on different electron transporting materials.

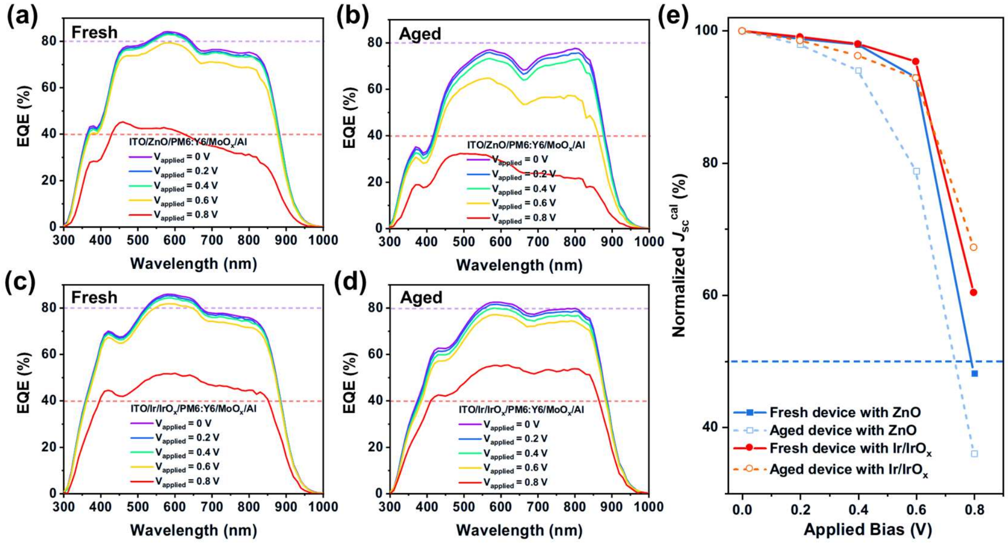

Supplementary Fig. 26. The bias-dependent EQE characterization. The bias-dependent EQE curves of fresh and aged PM6:Y6 device on (a, b) ZnO and (c, d) Ir/IrO$_x$, respectively. (e) The evolution of normalized J_{sc}^{cal} with applied bias, which was extracted from bias-dependent EQE curves of fresh and aged PM6:Y6 devices on different electron transporting materials.

Supplementary Table 9. The data of bias-dependent EQE characterization. The summary of calibration J_{sc} extracted from bias-dependent EQE spectra.

**Bias (V)**		**0**	**0.2**	**0.4**	**0.6**	**0.8**
**Fresh**	**J_{sc}^{cal} (mA/cm^2)**	24.63	24.33	24.12	22.89	11.87
**ZnO**	**Normalized (%)**	100	98.78	97.93	92.94	48.19
**Aged**	**J_{sc}^{cal} (mA/cm^2)**	22.88	22.40	21.51	18.03	8.25
**ZnO**	**Normalized (%)**	100	97.90	94.01	78.80	36.06
**Fresh**	**J_{sc}^{cal} (mA/cm^2)**	25.19	24.96	24.69	24.02	15.20
**Ir/IrO$_x$**	**Normalized (%)**	100	99.09	98.02	95.36	60.34
**Aged**	**J_{sc}^{cal} (mA/cm^2)**	24.75	24.39	23.82	22.98	16.63
**Ir/IrO$_x$**	**Normalized (%)**	100	98.55	96.24	92.85	67.19

Section 7. The morphology evolution of BHJ films on different interlayers

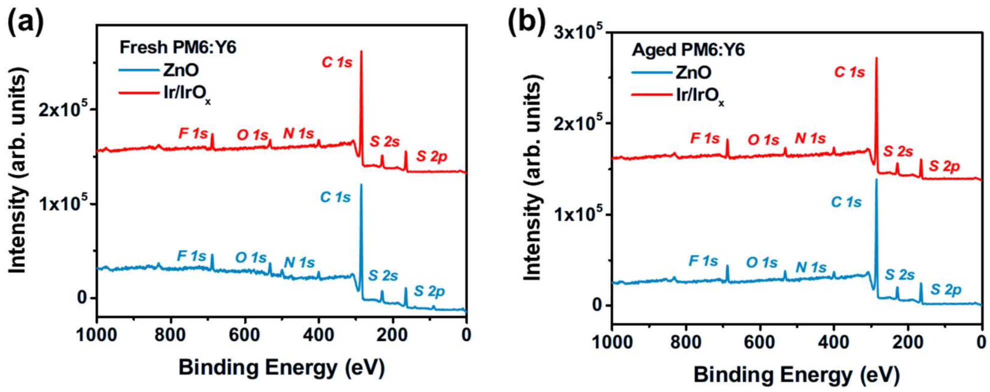

Supplementary Fig. 27. XPS plots of BHJ films. XPS characteristics of (a) fresh and (b) aged thin BHJ film on different electron transporting materials. The thickness of BHJ films is 10 nm.

Supplementary Table 10. The element ratio extracted from the XPS plots. The summary of element ratio in fresh and aged 10 nm BHJ films on different electron transporting materials.

BHJ	Fresh/aged	Interface	C (%)	O (%)	N (%)	S (%)	F (%)
PM6:Y6 (10 nm)	Fresh	ZnO	82.43	3.30	3.29	8.26	2.71
		Ir/IrO_x	82.19	2.66	3.62	8.94	2.58
	Aged	ZnO	84.03	1.95	2.77	8.65	2.60
		Ir/IrO_x	82.80	2.52	3.48	8.43	2.76

Supplementary Table 11. The *O 1s* signal of ZnO films. The statistic of XPS data extracted from the XPS plot of *O 1s* in ZnO films.

Materials	Properties	*O 1s*		
		O-H	O-Vacancy	Zn-O
Fresh ZnO	Position(eV)	531.83	531.00	530.11
	Area (%)	27.85	25.57	46.58
Aged ZnO	Position(eV)	532.18	531.28	530.33
	Area (%)	28.43	30.47	41.10

Supplementary Table 12. **The *O 1s* signal of Ir/IrO$_x$ films.** The statistic of XPS data extracted from the XPS plot of *O 1s* in Ir/IrO$_x$.

Materials	Properties	*O 1s*	
		O-H	Ir-O
Fresh Ir/IrO$_x$	Position(eV)	532.79	531.24
	Area (%)	87.70	12.30
Aged Ir/IrO$_x$	Position(eV)	532.82	531.16
	Area (%)	87.79	12.21

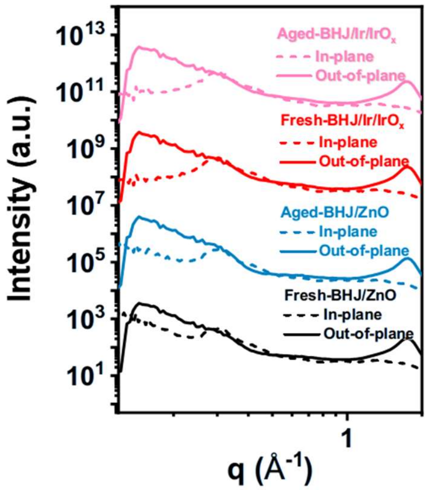

Supplementary Fig. 28. Line-cut of profiles of GIWAXS. The line-cut of profiles along the IP and OOP direction of 2D-GIWAXS patterns.

Supplementary Table 13. The summary of CCL. The statistic of parameters extracted from GIWAXS characterization. $\Delta CCL = (CCL_{Aged} - CCL_{Fresh}) / CCL_{Fresh} \times 100\%$, the CCL_{Fresh} and the CCL_{Aged} are CCL parameters of fresh and aged BHJ films, respectively.

Interface	Fresh/aged	FWHM (Å^{-1})	CCL (Å)	ΔCCL (%)
ZnO	Fresh	0.2872	19.68	-7.88
	Aged	0.3117	18.13	
Ir/IrO_x	Fresh	0.2189	25.82	-0.77
	Aged	0.2206	25.62	

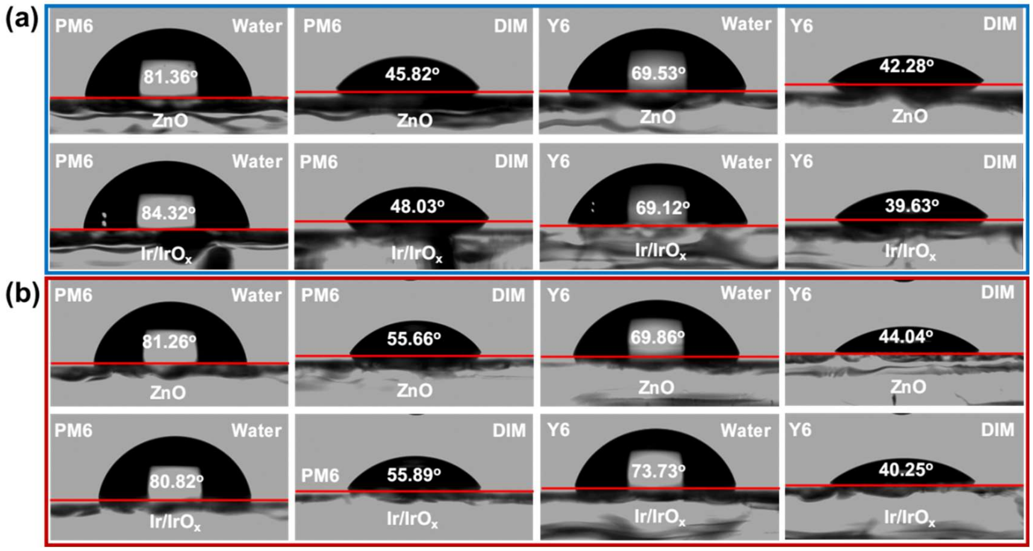

Supplementary Fig. 29. Contact angle characterizations. Contact angle photographs of (a) fresh and (b) aged PM6 and Y6 films deposited on different electron transporting materials.

Supplementary Table 14. The summary of Flory-Huggins parameters. The statistic of surface energy (γ_s) and Flory-Huggins parameters (χ) of fresh and aged active layers on different electron transporting materials. $\Delta\chi = (\chi_{Aged} - \chi_{Fresh}) / \chi_{Fresh} \times 100\%$, the χ_{Fresh} and the χ_{Aged} are χ parameters of fresh and aged BHJ films, respectively.

Interface	Fresh/aged	Materials	γ_s (mN/m)	χ (*K)	$\Delta\chi$ (%)
ZnO	Fresh	PM6	40.22	0.22	182
		Y6	46.43		
	Aged	PM6	36.03	0.62	
		Y6	46.11		
Ir/IrO_x	Fresh	PM6	38.31	0.47	11
		Y6	47.29		
	Aged	PM6	36.15	0.52	
		Y6	45.33		

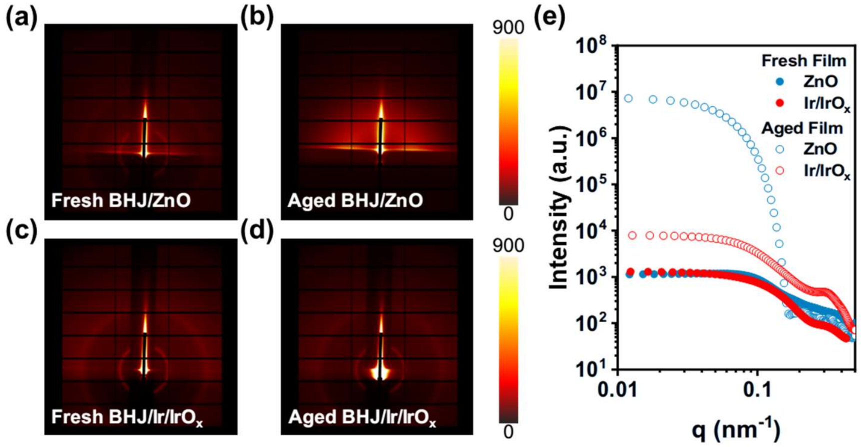

Supplementary Fig. 30. GISAXS characterizations. (a-d) 2D GISAXS patterns and (e) fitting curves of fresh and aged films on different electron transporting materials.

Supplementary Table 15. The summary of domain size. The statistic of domain size (d) of phase separation in BHJ films on different electron transporting materials. $\Delta d = (d_{Aged} - d_{Fresh}) / d_{Fresh} \times 100\%$, the d_{Fresh} and the d_{Aged} are domain size of phase separation in fresh and aged BHJ films, respectively.

**Interface**	d_{Fresh} **(nm)**	d_{Aged} **(nm)**	Δd **(%)**
ZnO	10.22	28.46	178
Ir/IrO_x	12.82	13.07	2

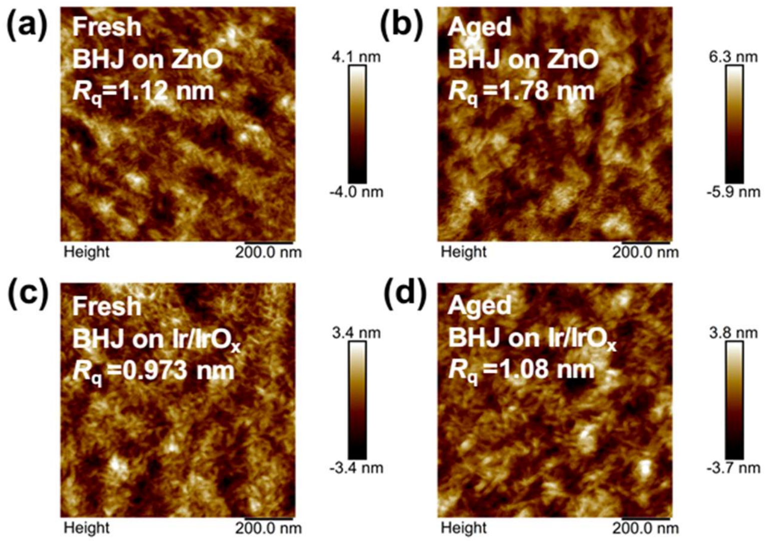

Supplementary Fig. 31. AFM morphology of BHJ films. AFM topography of (a) fresh PM6:Y6 film on ZnO, (b) aged PM6:Y6 film on ZnO, (c) fresh PM6:Y6 film on Ir/IrO$_x$ and (d) aged PM6:Y6 film on Ir/IrO$_x$.

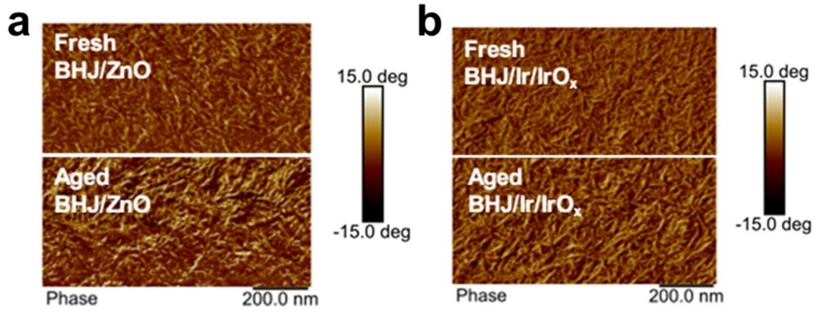

Supplementary Fig. 32. AFM phase patterns of BHJ films. AFM phase images of (a) fresh and aged PM6:Y6 film on ZnO and (b) fresh and aged PM6:Y6 film on Ir/IrO$_x$.

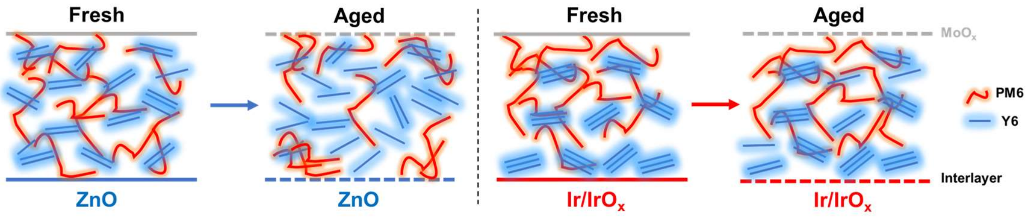

Supplementary Fig. 33. The schematic diagram. The schematic diagram of morphology evolution in BHJ films during aging process. The red fiber-like line and the blue line represents the PM6 molecule and Y6 molecule, respectively.

Section 8. Device stability under extreme conditions

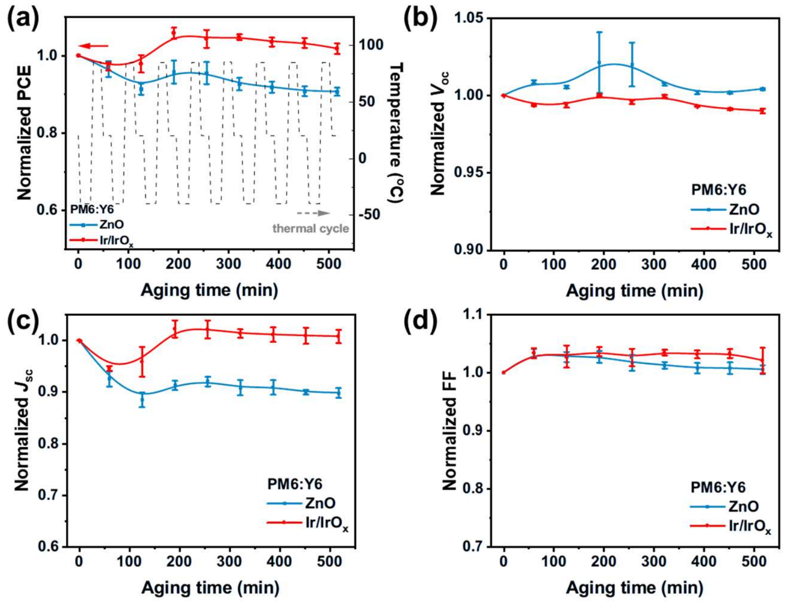

Supplementary Fig. 34. The attenuation of device parameters during thermal cycle. The evolution plots of (a) PCE, (b) V_{oc}, (c) J_{sc} and (d) FF of PM6:Y6 devices under thermal circulation (ISOS-T-3). The error bar is the standard deviation and it is calculated based on 6 devices for each interfacial material. The short-dashed line in Supplementary Fig. 29a represents the trend of temperature in thermal-cycle process.

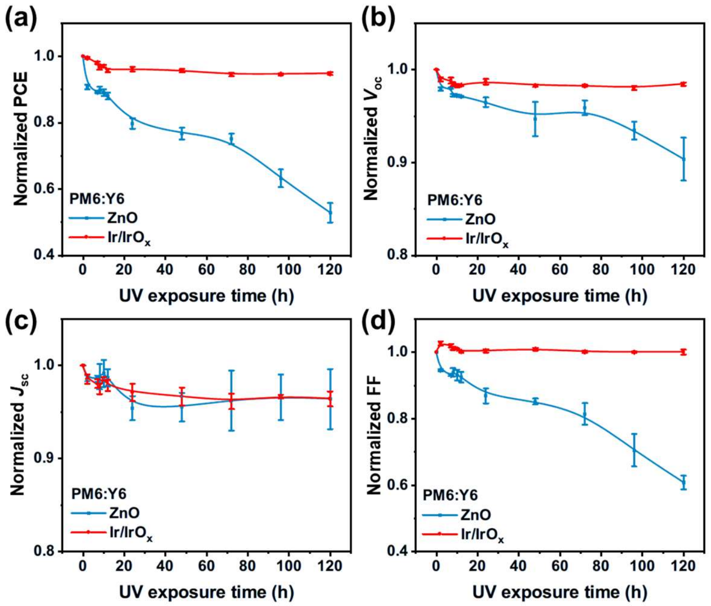

Supplementary Fig. 35. The attenuation of device parameters under UV-irradiation. The evolution plots of (a) PCE, (b) V_{oc}, (c) J_{sc} and (d) FF in PM6:Y6 devices under UV-irradiation (365 nm). The error bar is the standard deviation and it is calculated based on 6 devices for each interfacial material.

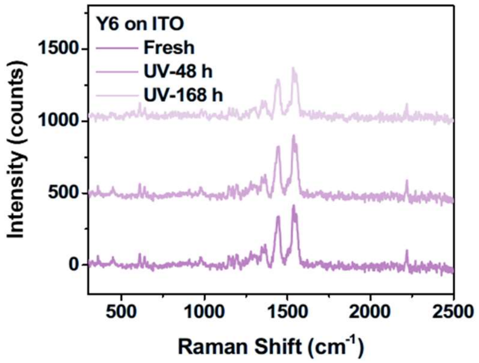

Supplementary Fig. 36. The evolution of Raman spectra. The Raman spectra of Y6 on ITO during the UV-irradiation aging.

Supplementary References

1 Xiao, C. *et al.* Weak affinity for CO of platinum group metal nanoparticles supported on partially reduced iron oxides. *Journal of Nanoparticle Research* **15** (2013).

2 Liang, M., Wang, X., Liu, H., Liu, H. & Wang, Y. Excellent catalytic properties over nanocomposite catalysts for selective hydrogenation of halonitrobenzenes. *Journal of Catalysis* **255**, 335-342 (2008).

3 Wang, Y. R., J.; Deng, K.; Gui, L.; Tang, Y. . Preparation of Tractable Platinum, Rhodium, and Ruthenium Nanoclusters with Small Particle Size in Organic Media. *Chem. Mater.* **12**, 1622-1627 (2000).
