# Supplementary material for: Lifetime over 10000 hours for organic solar cells with Ir/IrOx electron-transporting layer
Source: Nat Commun. 2023 Mar 4;14:1241. doi: 10.1038/s41467-023-36937-8 (PMC9985642; doi:10.1038/s41467-023-36937-8)
Supplement: Supplementary file 2 — Solar Cells Reporting Summary [file 41467_2023_36937_MOESM2_ESM.pdf]

nature research

Corresponding author(s): Huiqiong Zhou

Solar Cells Reporting Summary

Nature Research wishes to improve the reproducibility of the work that we publish. This form is intended for publication with all accepted papers reporting the characterization of photovoltaic devices and provides structure for consistency and transparency in reporting. Some list items might not apply to an individual manuscript, but all fields must be completed for clarity.

For further information on Nature Research policies, including our data availability policy, see Authors & Referees.

Experimental design

Please check: are the following details reported in the manuscript?

1. Dimensions

Item	Yes/No	Details
Area of the tested solar cells	☒ Yes ☐ No	The active area of devices was 0.0256 cm2.
Method used to determine the device area	☒ Yes ☐ No	The active area of the solar cells was defined by an opaque metal mask with square aperture of the area of 0.0256 cm2.

2. Current-voltage characterization

Item	Yes/No	Details
Current density-voltage (J-V) plots in both forward and backward direction	☐ Yes ☒ No	Organic solar cells do not have hysteresis problem.
Voltage scan conditions<br>*For instance: scan direction, speed, dwell times*	☒ Yes ☐ No	The voltage scan rate and scan step were 20 mV s-1 and 20 mV for devices.
Test environment<br>*For instance: characterization temperature, in air or in glove box*	☒ Yes ☐ No	The J-V characteristic was performed at room temperature in N2-filled glovebox.
Protocol for preconditioning of the device before its characterization	☐ Yes ☒ No	No preconditioning was applied.
Stability of the J-V characteristic<br>*Verified with time evolution of the maximum power point or with the photocurrent at maximum power point; see ref. 7 for details.*	☒ Yes ☐ No	The operating stability of devices were performed at the MPP and it could be found in Fig. 4c and Supplementary Fig. 21-23.

3. Hysteresis or any other unusual behaviour

Item	Yes/No	Details
Description of the unusual behaviour observed during the characterization	☐ Yes ☒ No	No unusual behaviour was found in this research.
Related experimental data	☐ Yes ☒ No	No related experimental data.

4. Efficiency

Item	Yes/No	Details
External quantum efficiency (EQE) or incident photons to current efficiency (IPCE)	☒ Yes ☐ No	See Fig. 2f in manuscript.
A comparison between the integrated response under the standard reference spectrum and the response measure under the simulator	☒ Yes ☐ No	The integrated Jsc values from the EQE curves in Fig. 2f matched well with those measured from the J-V curves.
For tandem solar cells, the bias illumination and bias voltage used for each subcell	☐ Yes ☒ No	No tandem solar cell was fabricated in this research.

5. Calibration

Item	Yes/No	Details
Light source and reference cell or sensor used for the characterization	☒ Yes ☐ No	The J–V characteristic was performed by the solar simulator (SS-F5-3A, Enlitech) along with AM 1.5G spectra which intensity was calibrated by the certified standard silicon solar cell (SRC-2020, Enlitech) at 100 mW/cm2.
Confirmation that the reference cell was calibrated and certified	☒ Yes ☐ No	The reference cell (SRC-2020, Enlitech)was certified.

Calculation of spectral mismatch between the reference cell and the devices under test

- [ ] Yes
- [x] No

No spectral mismatch calculation was performed in our lab.

6. Mask/aperture

Size of the mask/aperture used during testing

- [x] Yes
- [ ] No

The area of the mask is 0.0256 cm2.

Variation of the measured short-circuit current density with the mask/aperture area

- [ ] Yes
- [x] No

It was not found in this research.

7. Performance certification

Identity of the independent certification laboratory that confirmed the photovoltaic performance

- [ ] Yes
- [x] No

It was not necessary in this research.

A copy of any certificate(s)
Provide in Supplementary Information

- [ ] Yes
- [x] No

It was not necessary in this research.

8. Statistics

Number of solar cells tested

- [x] Yes
- [ ] No

For the J-V characteristics, over 8 devices were tested for each condition. In the shelf-stability measurement, six devices were tested. In the thermal stability and MPP stability, five and three devices were tested respectively.

Statistical analysis of the device performance

- [x] Yes
- [ ] No

See Supplementary Table 5, 6 and 8 in supplementary information.

9. Long-term stability analysis

Type of analysis, bias conditions and environmental conditions
For instance: illumination type, temperature, atmosphere humidity, encapsulation method, preconditioning temperature

- [x] Yes
- [ ] No

For the thermal aging test, devices were placed on the hotplate heating at 60℃ in N2-filled glovebox, respectively. For the thermal-circulation stability, devices were placed in a vacuum chamber with the standard ISOS-T-3. For the UV-irradiation aging test, devices were placed under the 365 nm UV light.
